# Molecular Basis for Coordinating Transcription Termination with Noncoding RNA Degradation

**DOI:** 10.1016/j.molcel.2014.05.031

**Published:** 2014-08-07

**Authors:** Agnieszka Tudek, Odil Porrua, Tomasz Kabzinski, Michael Lidschreiber, Karel Kubicek, Andrea Fortova, François Lacroute, Stepanka Vanacova, Patrick Cramer, Richard Stefl, Domenico Libri

**Affiliations:** 1 Centre de Génétique Moléculaire, CNRS UPR3404, 91190 Gif sur Yvette, France; 2 CEITEC-Central European Institute of Technology, Masaryk University, Brno 62500, Czech Republic; 3 National Centre for Biomolecular Research, Faculty of Science, Masaryk University, Brno 62500, Czech Republic; 4 Gene Center Munich and Department of Biochemistry, Center for Integrated Protein Science CIPSM, Ludwig-Maximilians-Universität München, 81377 Munich, Germany

## Abstract

The Nrd1-Nab3-Sen1 (NNS) complex is essential for controlling pervasive transcription and generating sn/snoRNAs in *S. cerevisiae*. The NNS complex terminates transcription of noncoding RNA genes and promotes exosome-dependent processing/degradation of the released transcripts. The Trf4-Air2-Mtr4 (TRAMP) complex polyadenylates NNS target RNAs and favors their degradation. NNS-dependent termination and degradation are coupled, but the mechanism underlying this coupling remains enigmatic. Here we provide structural and functional evidence demonstrating that the same domain of Nrd1p interacts with RNA polymerase II and Trf4p in a mutually exclusive manner, thus defining two alternative forms of the NNS complex, one involved in termination and the other in degradation. We show that the Nrd1-Trf4 interaction is required for optimal exosome activity in vivo and for the stimulation of polyadenylation of NNS targets by TRAMP in vitro. We propose that transcription termination and RNA degradation are coordinated by switching between two alternative partners of the NNS complex.

## Highlights

• The Nrd1 CTD interaction domain (CID) recognizes a CTD mimic in Trf4
• The CID interacts with RNAPII and Trf4 in a mutually exclusive manner
• Architecture of the interactions between the NNS complex, the exosome, and TRAMP
• The interaction of Nrd1 with Trf4 stimulates the polyadenylation activity of TRAMP

## Graphical Abstract

**Figure:**
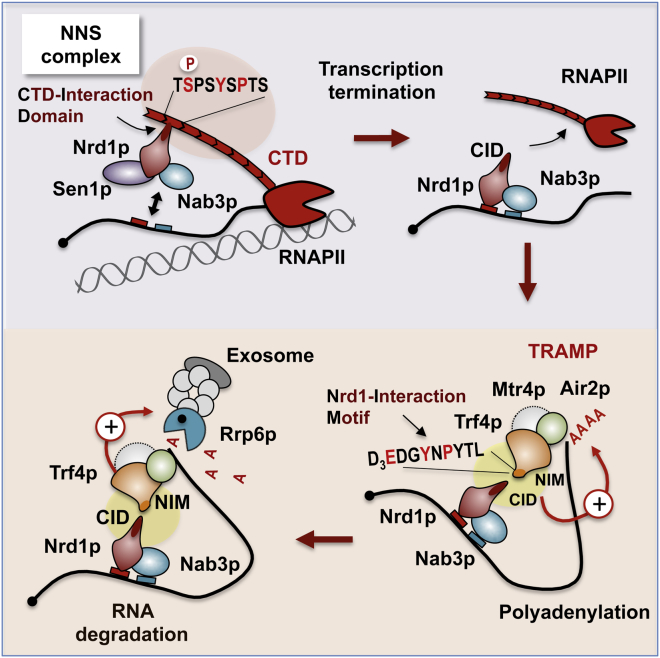

## Teaser

The Nrd1-Nab3-Sen1 complex controls pervasive transcription in yeast by terminating transcription of cryptic transcripts and directing these RNAs to degradation by the exosome. Tudek et al. show that the same domain of Nrd1p mediates mutually exclusive interactions with RNAPII or the exosome cofactor TRAMP to coordinate termination and RNA degradation.

## Introduction

The ubiquitous presence of transcribing polymerases in the genome is a potential risk to the cell, as it can hamper the appropriate expression of canonical genes by interfering with their transcription (Jensen et al., 2013). Pervasive transcription is controlled at the level of transcription termination and RNA degradation, which can be coupled in *S. cerevisiae*. The main actors of this quality control pathway are the Nrd1-Nab3-Sen1 (NNS) transcription termination complex, the nuclear exosome, and the TRAMP complex (for recent reviews, see Porrua and Libri, 2013a, Jensen et al., 2013). The NNS complex is required for transcription termination of a large fraction of noncoding RNAs (ncRNAs) transcribed by RNA polymerase II (RNAPII), essentially CUTs (cryptic unstable transcripts), snRNAs, and snoRNAs (Steinmetz et al., 2001, Arigo et al., 2006, Thiebaut et al., 2006). CUTs are short lived in wild-type yeast and are largely nonfunctional, although in a few cases their transcription has been shown to regulate gene expression (Kuehner and Brow, 2008, Thiebaut et al., 2008). Transcripts terminated by the NNS pathway are polyadenylated by the TRAMP complex and targeted by the nuclear exosome for degradation (in the case of CUTs) or 3′ end trimming (in the case of snRNAs and snoRNAs).

The exosome is composed of a ring-shaped core to which two catalytic subunits, Dis3p and Rrp6p, associate. The two enzymes are 3′-5′ exonucleases, and Dis3p is also endowed with endonuclease activity (Chlebowski et al., 2013). Because the central channel of the ring that drives the substrate toward the catalytic subunit Dis3p is only wide enough to accommodate single-stranded RNA, it has been proposed that the presence of an unstructured region of at least 30 residues is required for degradation (Chlebowski et al., 2013). Rrp6p only associates with the nuclear form of the exosome and has overlapping and complementary roles to Dis3p in RNA degradation (Gudipati et al., 2012).

The TRAMP complex is an important cofactor of the exosome that is required for the efficient processing and degradation of a variety of RNAs produced by the three yeast RNA polymerases (Wyers et al., 2005, San Paolo et al., 2009, Wlotzka et al., 2011, Kadaba et al., 2004). TRAMP is composed of a poly(A) polymerase (Trf4p or Trf5p), a zinc knuckle RNA-binding protein (Air1p or Air2p), and the DExH-box RNA helicase Mtr4p (LaCava et al., 2005, Vanácová et al., 2005, Wyers et al., 2005). Polyadenylation of exosome substrates by TRAMP favors their degradation (Callahan and Butler, 2010, Kadaba et al., 2004, LaCava et al., 2005, Rougemaille et al., 2007, Vanácová et al., 2005, Wyers et al., 2005), and it has been proposed that poly(A) tails added by TRAMP provide the unstructured extensions that allow threading of structured substrates through the central channel of the exosome ring. It has also been shown that TRAMP stimulates exosome and Rrp6p activity independently of polyadenylation (Wyers et al., 2005, Callahan and Butler, 2010, Rougemaille et al., 2007), although the mechanistic details of this stimulation are still unclear.

The NNS complex is composed of the RNA-binding proteins Nrd1p and Nab3p and the superfamily I helicase Sen1p. Binding of the Nrd1-Nab3 complex to specific motifs on the nascent RNA constitutes the essential readout of transcription termination signals (Creamer et al., 2011, Porrua et al., 2012, Wlotzka et al., 2011). The actual termination step is most likely operated by Sen1p that interacts with the Nrd1-Nab3 complex (Hazelbaker et al., 2013, Porrua and Libri, 2013b). The NNS complex has been shown to associate with TRAMP and the exosome, which is thought to favor degradation (Vasiljeva and Buratowski, 2006), although the molecular and mechanistic details of the interaction between the NNS complex, the exosome, and the TRAMP are not well understood.

Nrd1p interacts with the C-terminal domain (CTD) of the largest subunit of RNAPII via a CTD interacting domain (CID). The CID recognizes heptapeptide (YSPTSPS) repeats in the CTD that are phosphorylated on the serine at position five (Ser5P) (Kubicek et al., 2012, Mayer et al., 2012, Vasiljeva et al., 2008). Because this modification mark predominates early in transcription, when NNS-dependent termination preferentially occurs (Buratowski, 2009, Gudipati et al., 2008, Jenks et al., 2008, Steinmetz et al., 2006a), the CID-CTD interaction is believed to determine the regional specificity of termination (Gudipati et al., 2008, Vasiljeva et al., 2008). However, previous studies did not detect significant termination defects in a *nrd1ΔCID* background (Vasiljeva et al., 2008). Surprisingly, RNAs produced by NNS termination were found to be stabilized in *nrd1ΔCID* cells, suggesting that the CID domain might favor degradation/processing by the exosome (Kubicek et al., 2012, Vasiljeva et al., 2008).

Here we analyze the role of the Nrd1p CID in transcription termination and in promoting RNA degradation/processing by the nuclear exosome. We detected widespread termination defects at NNS targets and defective recruitment of Nrd1p to elongation complexes upon deletion of the CID. Surprisingly, we discovered that the CID also mediates the interaction between the NNS complex and TRAMP by recognizing in Trf4p a CTD mimic that we dubbed NIM (for Nrd1 interaction motif). We solved the solution structure of the interaction surface, and we show that the interactions of Nrd1p with TRAMP and RNAPII are mutually exclusive. Importantly, we demonstrate that the Nrd1p-Trf4p interaction stimulates the polyadenylation activity of TRAMP in vitro, suggesting that the CID contributes to efficient degradation of exosome substrates by facilitating TRAMP recruitment and function. Our results demonstrate the existence of two alternative forms of the NNS complex: one associated with RNAPII and functioning in termination and the other associated with TRAMP and promoting RNA degradation.

## Results

### The Nrd1p CID Domain Plays a Role in NNS-Dependent Transcription Termination

**Figure 1:**
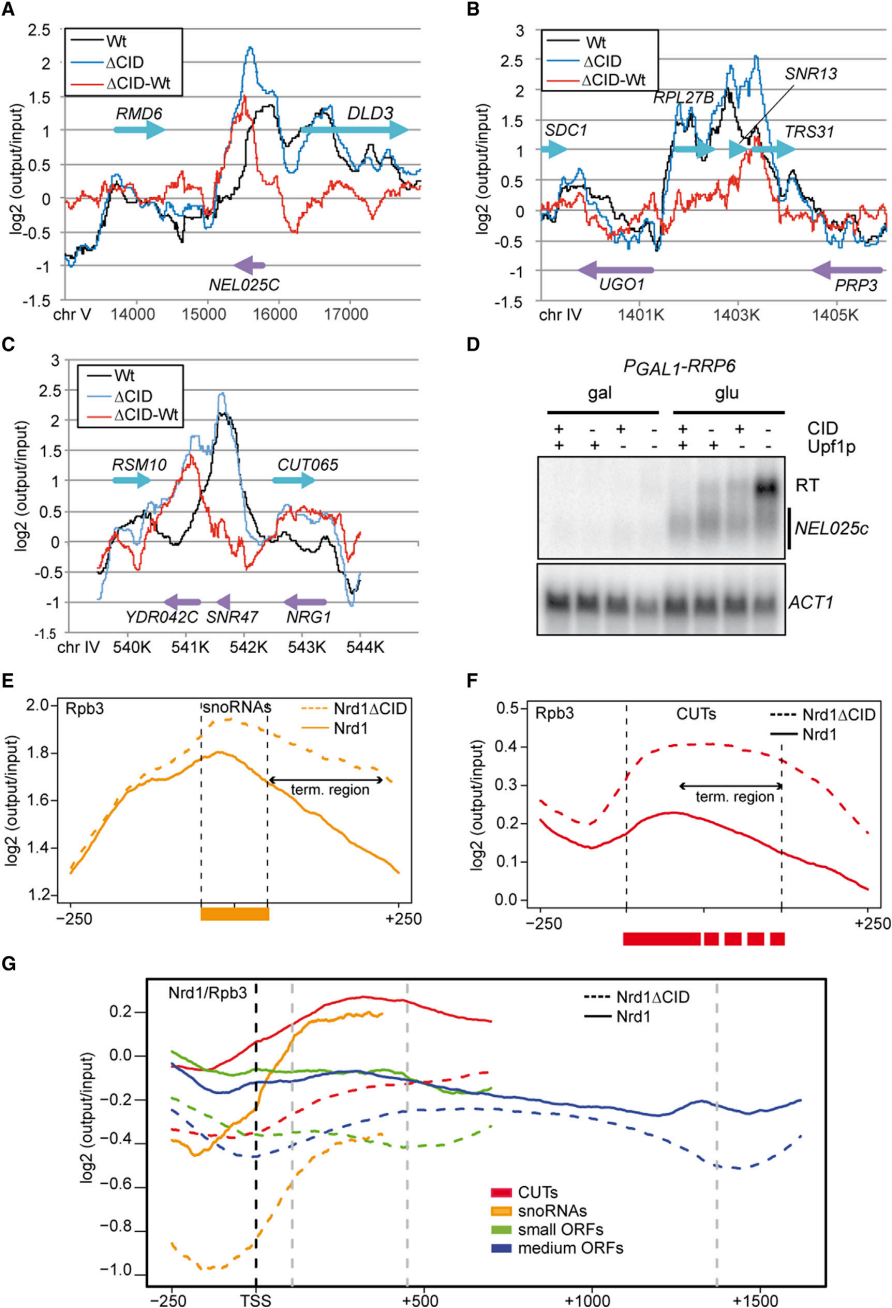
Effect of CID Deletion on RNAPII and Nrd1p Occupancy Determined by ChIP-Chip at NNS Targets (A–C) Rpb3p-TAP occupancy is plotted at the *NEL025c* (A), *SNR13* (B), and *SNR47* (C) loci in a wild-type (black) or an *nrd1ΔCID* (blue) strain as indicated. The difference between the two signals (ΔCID-WT) is also plotted in red. The position of the relevant features on the W or C strand is indicated by blue and violet arrows, respectively. The signal (log2 ratio) is normalized to its genome-wide median level. (D) Northern blot analysis of *NEL025c* transcripts in the presence and absence of the Nrd1p CID and in NMD *Δupf1* mutant cells. In this experiment, Rrp6p was metabolically depleted using the glucose-repressible *GAL1* promoter as indicated. (E and F) Metagene analysis of RNAPII distribution for *NRD1* and *nrd1ΔCID* strains at snoRNAs (E) and CUTs (F). All features have been scaled and aligned either to the coordinates of the mature transcript (snoRNAs) or to the annotation based on tiling array analyses (CUTs; Xu et al., 2009). Alignment borders are indicated by gray dotted lines. The approximate range of termination for CUTs is indicated by a double arrow. (G) Metagene distribution of Nrd1p occupancy for the different classes of features as indicated, in the presence (plain lines) or absence (dotted lines) of the CID. All signals are normalized to Rpb3p occupancy to limit biases due to differences in transcription levels. Raw Nrd1p signals for all classes are shown in Figure S1. Features have been scaled and aligned as in (E) and (F) and in Figure S1.

To assess the role of the Nrd1p CID in the function of the NNS complex, we reexamined whether this domain is required for efficient transcription termination by the NNS pathway. We compared the RNAPII distribution by chromatin immunoprecipitation (ChIP) and tiling arrays in wild-type and *nrd1ΔCID* cells. Upon deletion of the CID, we observed persistent presence of RNAPII downstream of many genes, such as the CUT *NEL025C*, *SNR13*, *SNR47*, and *CUT065* (Figures 1A–1C), indicating transcriptional readthrough. These findings were confirmed by northern blot analyses (Figures 1D and S2A, available online) and showed that in some cases (e.g., *NEL025c*) poor detection of the readthrough transcripts is due to the combined nuclear and cytoplasmic degradation of these species. Indeed, readthrough transcripts become prominent in cells in which both the nuclear exosome and the cytoplasmic nonsense-mediated decay (NMD) degradation pathways are defective (i.e, in a Rrp6p-depleted, *Δupf1* mutant; Figure 1D).

Metagene analyses suggested that readthrough occurs at the majority of NNS targets, such as CUTs and snoRNAs (Figures 1E and 1F; see also Supplemental Experimental Procedures). Readthrough also occurs at small open reading frames (ORFs; <500 bp; Figure S1A) that have been shown to be partially NNS dependent (Steinmetz et al., 2006b) and possibly at a subset of larger ORFs (Figure S1B), although in both cases it is not possible to clearly distinguish bona fide readthrough events from failure to terminate antisense transcription that is frequently observed at the 3′ end of ORFs (Neil et al., 2009, Xu et al., 2009). Consistent with this notion, genes with no detected antisense transcripts like *RMD6*, *DLD3*, *TRS31*, *UGO1*, and *PRP3* display no significant readthrough (Figures 1A and 1B).

We compared the genome-wide chromatin distribution of Nrd1p in the presence and the absence of the CID by ChIP-chip analysis, normalizing to transcription levels as defined by RNAPII occupancy in both strains (Figure 1G; for the non-normalized Nrd1p occupancy, see Figure S1C). Metagene analyses for the four distinct classes of features showed that wild-type Nrd1p is recruited to higher levels at CUTs and snoRNAs genes, but also at the 5′ end of ORFs as previously reported (Kim et al., 2010, Mayer et al., 2010), although the 5′ end recruitment peak was attenuated by RNAPII normalization (Figures 1G and S1D). Importantly, ablation of the CID domain affected recruitment in all instances, although higher Nrd1p occupancy persisted at CUTs relative to other features in *nrd1ΔCID* cells, presumably because of RNA-mediated recruitment. As expected, no effects of CID deletion were observed at tRNA genes (Figure S1E). Altogether, these results demonstrate that the CID is required for efficient termination at NNS-dependent targets.

### Nrd1p CID Recognizes the Trf4p Component of the TRAMP Complex

Consistent with previous reports (Kubicek et al., 2012, Vasiljeva et al., 2008), we observed that the levels of the *NEL025C* CUT, pre-snR13, and pre-snR47 were increased upon deletion of Nrd1 CID (Figure S2A). Stabilization was stronger when the nuclear degradation machinery was compromised in *rrp6* catalytic mutants (Figure S2A), presumably because of partial redundancy in the degradation pathways. Interestingly, we also detected stabilization of some degradation intermediates derived from the U4 and U5 snRNAs (Figure S2B), suggesting a more general CID requirement for optimal activity of Rrp6-exosome.

**Figure 2:**
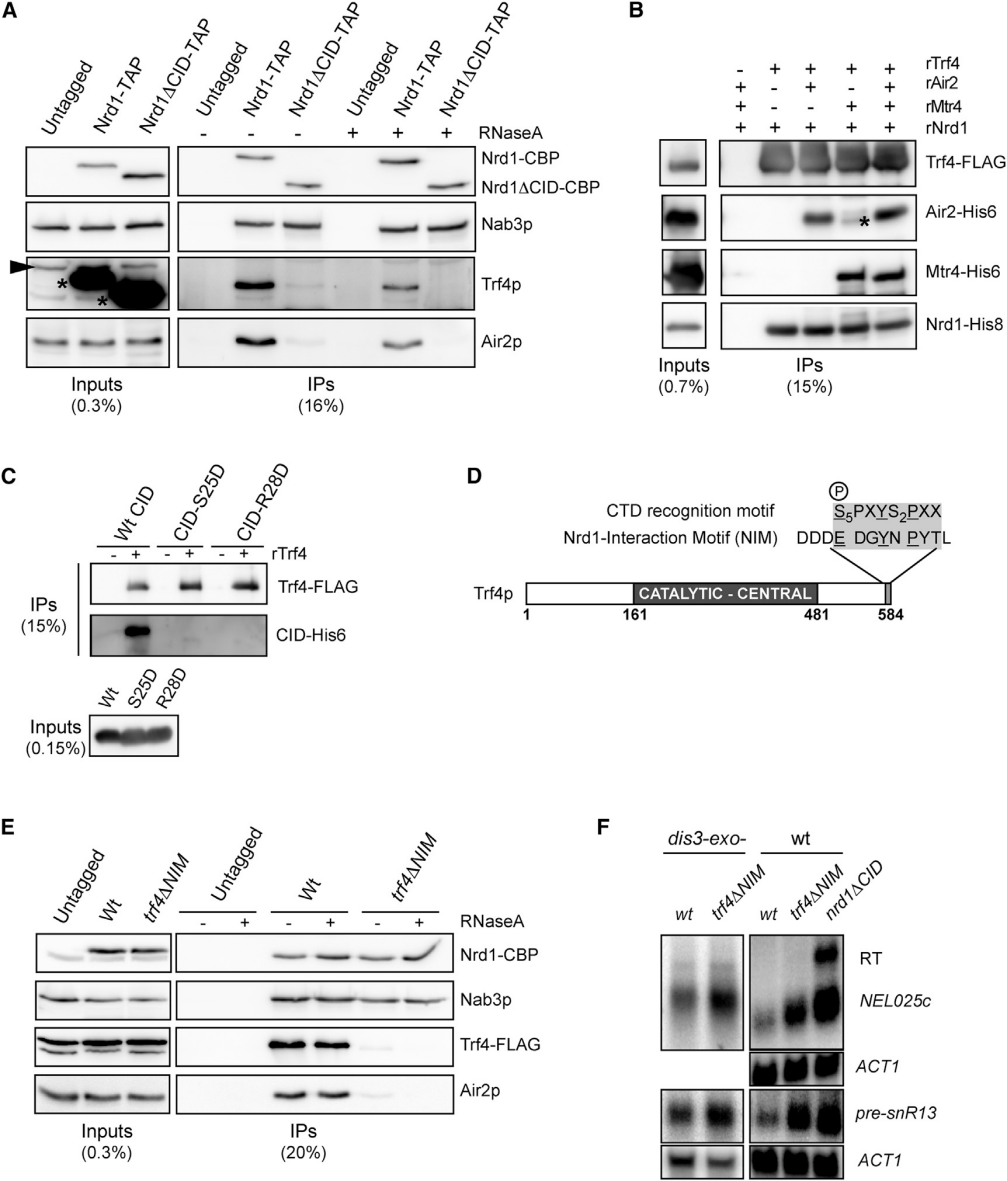
Direct Interaction between Trf4p and Nrd1p Is Mediated by the CID (A) Western blot analysis of Nrd1p-TAP and Nrd1ΔCIDp-TAP immunopurified complexes (IP). Samples were eluted by cleavage with the TEV protease. The indicated proteins were detected with specific antibodies, except for Nrd1p, which was detected with an anti-CBP antibody. The Trf4 signal in the input (indicated by an arrowhead) is partially overlapping with the Nrd1-TAP signal (denoted by an asterisk). The fraction of extract and immunoprecipitated material that is loaded on the gel is indicated. (B) Immunoblot analysis of pull-down experiments performed with recombinant Trf4-FLAG as bait and *E. coli* extracts expressing recombinant His-tagged Air2, Mtr4, or Nrd1 as indicated. Immunoprecipitations were performed in the presence of RNase. An asterisk indicates a degradation fragment of Mtr4-His_6_. (C) Immunoblot analysis as in (B) using recombinant Trf4-FLAG and recombinant CID-His_6_ or CID mutant derivatives (rCID-S25D-His_6_ and CID-R28D-His_6_), defective for the interaction with the CTD. Proteins were detected with an antibody anti-His tag or anti-FLAG. (D) Scheme of Trf4p indicating the position and sequence of the NIM, compared to a CTD pattern containing the amino acids that mediate major contacts with the CID, including Ser5P (equivalent CTD and NIM regions are shaded; identical amino acids are underlined). Note the presence of a Ser5 phosphomimic (E) in the NIM. (E) Western blot analysis of Nrd1-TAP immunopurified complexes from a *TRF4* or *trf4ΔNIM* strain as in (A). (F) Stabilization of NNS targets in *trf4Δ*NIM cells. Analysis of *NEL025C* and pre-snR13 RNAs by northern blot in the presence or absence of the NIM, in an exosome-defective (Dis3p catalytic mutant, *dis3-exo*^*−*^; left panels) or an otherwise wild-type background (right panels). Note that the rightmost panels were more exposed than the other panels to visualize the poorly detectable *NEL025C* and pre-snR13 transcripts in a strain wild-type for the nuclear exosome. Stabilization values in *dis3-exo*^*−*^*/trf4ΔNIM* relative to *dis3-exo*^*−*^ are 1.9 ± 0.37 and 2.5 ± 0.56 for pre-snR13 and *NEL025c*, respectively (average and SD from three independent samples). Stabilization of pre-snR13 and *NEL025c* was consistently observed in *trf4ΔNIM* cells but could not be reliably quantified due to the low levels of these RNAs in a wild-type background.

Because the NNS complex has previously been shown to copurify with the exosome and TRAMP complexes (Vasiljeva and Buratowski, 2006), we considered that the CID could be involved in mediating such interactions. To address this question, we first performed coimmunoprecipitation assays with wild-type or ΔCID TAP-tagged Nrd1p. We consistently observed strong signals for the TRAMP components Trf4p and Air2p in Nrd1p immunoprecipitates. Strikingly, however, TRAMP signals could not be detected in the absence of the CID in ribonuclease (RNase)-treated extracts (Figure 2A), which was also reported by Heo et al. (2013) while this work was in progress. As expected, deletion of the CID did not affect the interaction between Nrd1p and Nab3p (Figure 2A).

In contrast to the strong TRAMP signals, we only detected weak signals for Rrp6p and Dis3p in Nrd1-TAP immunoprecipitates upon RNase treatment (Figure S3A). This suggests that the TRAMP complex is a major partner of the NNS complex relative to the exosome. In order to assess whether the interaction between Nrd1p and the TRAMP is direct, we performed pull-down experiments using *E. coli* extracts containing recombinant TRAMP components and Nrd1 (Figure 2B). We observed a robust, RNA-independent interaction between rNrd1 and rTrf4, even in the absence of rAir2 and rMtr4. Importantly, rNrd1ΔCID failed to interact with rTRAMP (Figure S3B), and recombinant isolated CID (rCID-His_6_) alone was efficiently pulled down by rTrf4 (Figure 2C). Taken together, these results demonstrate that Nrd1p recognizes the TRAMP complex via a direct interaction with Trf4p, the CID domain being necessary and sufficient for this interaction.

### The Nrd1p Interaction Motif Is a Short CTD-like Domain in Trf4p

Since the CID interacts with the RNAPII CTD and Trf4p, we considered that the same surface might be involved in the recognition of both targets. Consistent with this notion, two CID variants mutated at positions that are critical for binding to the Ser5P CTD (S25D and R28D; Kubicek et al., 2012) also failed to interact with rTrf4 (Figure 2C). Therefore, we surmised that the Nrd1p CID might recognize a CTD mimic in Trf4p. Because the CTD is intrinsically disordered, we restricted our search to the unstructured N- and C-terminal regions of Trf4p. We found a 9 aa motif at the very C-terminal end of Trf4p, which contains several residues that are critical in the CTD for the interaction with the CID, including a glutamate that could mimic the Ser5P in CTD repeats (Figures 2D and S3C).

To assess the role of this motif, we immunoprecipitated Nrd1-TAP from strains expressing a mutant variant of Trf4p lacking the last nine C-terminal amino acids and the two adjacent aspartic acid residues, which we surmised to be important for binding based on the CID structure (Kubicek et al., 2012). Despite identical steady-state levels of the wild-type and mutant Trf4p, the interaction with Nrd1p was abolished in the mutant, indicating that the C-terminal region is necessary (Figure 2E). Therefore, we dubbed this motif NIM, for Nrd1p interaction motif.

We set out to test whether the lack of interaction between Nrd1p and Trf4p contributes to the degradation/processing defects observed in the *nrd1-ΔCID* mutant (Figure S2). To this end, we analyzed by northern blot the effect of the NIM deletion on the levels of the *NEL025c* CUT and the *SNR13* precursor. As shown in Figure 2F, in *trf4ΔNIM* cells these NNS targets were stabilized, although to levels slightly lower than those in an *nrd1ΔCID* mutant. As for deletion of the CID, deletion of the NIM exacerbates the degradation/processing phenotype of exosome defective cells (*dis3-exo-*; Figure 2F). We did not observe any significant effect of the NIM deletion on termination based on the detection of readthrough transcripts or RNAPII ChIP (Figures 2F and S3E and data not shown), suggesting that the Nrd1p-Trf4p interaction is not required for transcription termination. Taken together, these results demonstrate that Nrd1p recognizes a CTD-like motif, NIM, in the Trf4p C-terminal region via its CID domain and that this interaction contributes to the role of the NNS complex in promoting RNA degradation by the Rrp6-exosome.

### The CID-CTD and CID-NIM Interactions Are Mutually Exclusive

**Figure 3:**
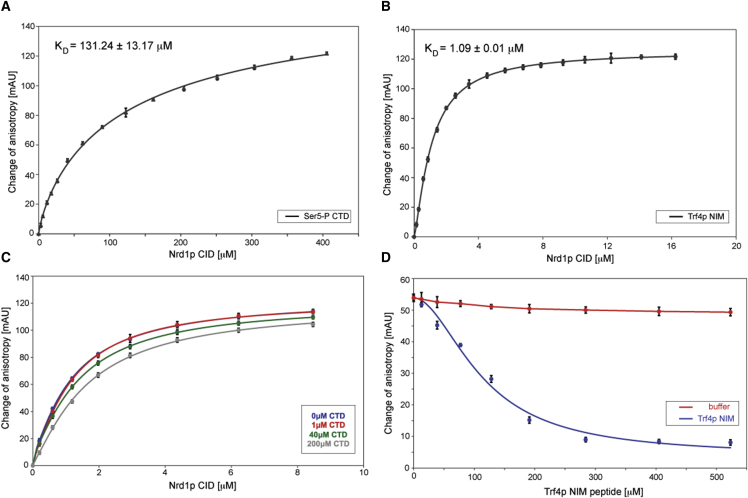
Fluorescence Anisotropy Analyses of Nrd1p CID Binding to Trf4 NIM and the CTD (A and B) Equilibrium binding of Nrd1p CID with Ser5P CTD (A) and Trf4p NIM (B) fluorescently labeled peptides monitored by fluorescence anisotropy (FA). Binding isotherms and dissociation constant (K_D_) are shown. (C) FA competition assays between Ser5P CTD and Trf4p NIM for binding to Nrd1p CID. Samples containing 10 nM FAM-labeled Trf4p NIM peptide and 0 μM (blue), 1 μM (red), 40 μM (green), or 200 μM (gray) of unlabeled Ser5P CTD were titrated with Nrd1p CID. Displacement of the binding isotherm with increasing concentration of Ser5P CTD indicates competition for binding to Nrd1p CID. (D) FA competition assays between pSer5 CTD and Trf4p NIM for binding to Nrd1p CID with a different experimental setup compared to (C). Preformed complex of 10 nM FAM-labeled Ser5P CTD and Nrd1p CID (120 μM final protein concentration) was titrated with different amounts of Trf4p NIM peptide (blue) or buffer (red) as a control. The decrease of fluorescence anisotropy reflects the disassembly of the Ser5P CTD-Nrd1p CID complex.

Previous studies have demonstrated that the CID of Nrd1p binds to a fragment consisting of two canonical CTD repeats with the Ser5P mark located in the upstream repeat (Kubicek et al., 2012). This phospho-CTD fragment binds Nrd1p CID with a K_D_ of ∼130 μM (Figure 3A). To assess the binding affinity of Nrd1p CID to the NIM, we performed a quantitative solution-binding assay using fluorescence anisotropy (FA) measurements. We found that Nrd1p CID binds NIM with an affinity roughly 100-fold stronger compared to the phospho-CTD peptide (a K_D_ of ∼1 μM; Figure 3B). To assess whether the interactions of the CID with CTD and Trf4p are mutually exclusive, we first performed titration of fluorescently labeled NIM peptides with Nrd1p CID in the absence or presence of unlabeled CTD (Figure 3C). The displacement of the binding isotherm in the presence of increasing CTD concentrations demonstrates that the CTD can outcompete Nrd1p CID from binding to the NIM, although the competition was only partially effective, as expected from the stronger affinity of the NIM for the CID. Importantly, increasing concentrations of unlabeled NIM could effectively disassemble a preformed Nrd1p CID-CTD complex in which the CTD was fluorescently labeled (Figure 3D). The latter competition assay was used to calculate a K_D_ of ∼120 μM for Nrd1p CID-Ser5P CTD complex (using a K_D_ of 1.08 μM for Nrd1p CID-Trf4p NIM interaction), which is similar to the noncompetitive binding assay (Figure 3B). In both titration experiments we observed no additional increase of anisotropy, indicating that the Nrd1p CID-CTD-NIM ternary complex is not formed. Altogether, the FA data showed that the interactions of Nrd1p CID with the CTD and the NIM are mutually exclusive.

### Solution Structure of Nrd1p CID Bound to Trf4p NIM

**Figure 4:**
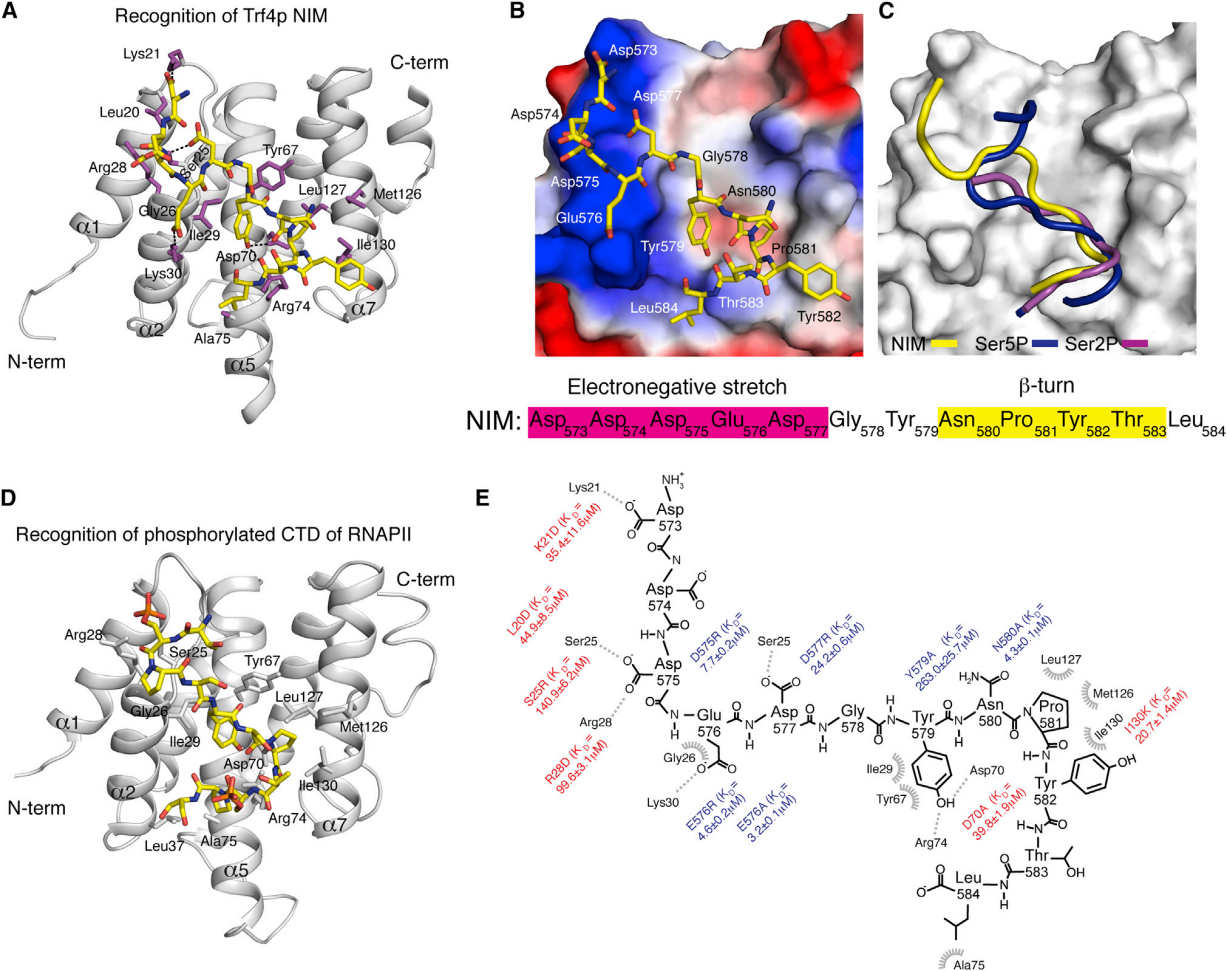
Recognition of the NIM Peptide by Nrd1p CID (A) Solution structure of Nrd1p CID bound to the NIM peptide. The NIM peptide is represented in yellow sticks (only nonhydrogen atoms are shown), and the protein is shown as a gray ribbon model. Protein residues that form hydrophobic contacts and putative hydrogen bonds to the NIM peptide are shown in magenta sticks. (B) Electrostatic surface representation of Nrd1p CID (electropositive in blue, electronegative in red, neutral in white) with the NIM peptide (represented in yellow sticks; only nonhydrogen atoms are shown). The upstream electronegative stretch of NIM interacts with the electropositive pocket of Nrd1p CID, while the β turn conformation formed by Asn580-Pro581-Tyr582-Thr583 docks in a hydrophobic pocket of Nrd1p CID. (C) Superposition of Nrd1p CID-Ser5P CTD (blue), Nrd1p CID-Trf4p NIM (yellow), and Pcf11p CID-Ser2P CTD (magenta) complexes, displaying only peptide ribbons on the surface of Nrd1p CID. The comparison highlights the β turn conformation recognition of CTDs and NIM by the CIDs. (D) Solution structure of Nrd1p CID bound to the Ser5P CTD peptide. The phospho-CTD peptide is represented in yellow sticks (only nonhydrogen atoms are shown), and the protein is shown as a gray ribbon model. Protein residues that form hydrophobic contacts and putative hydrogen bonds to the phospho-CTD peptide are shown in gray sticks. (E) Scheme showing contacts and energetics between the NIM peptide and Nrd1 CID. Equilibrium binding experiments with both the protein and peptide mutants (in red and blue, respectively) were monitored by FA (for the binding isotherms, see Figure S5). Other residues involved in the canonical CTD-CID interface were mutated previously (Kubicek et al., 2012, Vasiljeva et al., 2008). L20D mutant disrupts the hydrophobic contact with Phe17 and impairs the overall geometry of the α1-α2 loop that contributes to the interaction with the upstream electronegative stretch of NIM.

To understand how Trf4p is recognized by Nrd1p, we determined the solution structure of a reconstituted complex consisting of the CID (residues 1–153) of Nrd1p and a 12 aa NIM peptide (Asp573-Asp574-Asp575-Glu576-Asp577-Gly578-Tyr579-Asn580-Pro581-Tyr582-Thr583-Leu584) (Figures 4 and S4; Table 1). The structure of Nrd1p CID consists of eight α helices in a right-handed superhelical arrangement (Figure 4) and is similar to the structure of Nrd1p CID in the apo form (Vasiljeva et al., 2008) or bound to the phosphorylated CTD (Kubicek et al., 2012). The subtle differences originate from the extended interaction surface with Trf4p NIM, which involves loop α1-α2 and helices α2, α4, and α7 of Nrd1p CID (Figures 4A and S4A–S4G). Interestingly, [^1^H,^15^N] heteronuclear single quantum coherence (HSQC) titration experiments of Nrd1p CID revealed that the protein amide resonances are in fast or slow exchange regimes between their free and bound forms relative to NMR timescale, when titrated with the phosphorylated CTD or NIM, respectively (Figures S4C–S4E). This observation is in agreement with the fact that the two substrates differ in their binding affinities to the CID by two orders of magnitude as evidenced by the FA data.

**Table 1:** NMR and Refinement Statistics for the Nrd1p CID-Trf4p NIM Complex

Nrd1p CID-Trf4p NIM Complex	
**NMR Distance and Dihedral Constraints**	

Distance restraints	
Total NOEs	2,440
Intraresidue |i-j| = 0	602
Sequential |i-j| = 1	661
Medium range 1 < |i-j| < 5	700
Long range |i-j| ≥ 5	477
Hydrogen bonds	
Intermolecular distance restraints	54
Total dihedral angle restraints^a^	222

**Structure Statistics**^b^	

Violations (mean and SD)	
Number of distance restraint violations >0.5 Å	0
Number of dihedral angle restraint violations >15°	0
Maximum dihedral angle restraint violation (°)	6.67 ± 1.89
Maximum distance constraint violation (Å)	0.34 ± 0.12
Deviations from idealized geometry^b^	
Bond lengths (Å)	0.0035 ± 0.0001
Bond angles (°)	1.6 ± 0.02
Average pairwise r.m.s.d (Å)^b^	
Complex	
Heavy atoms	1.17 ± 0.08
Backbone atoms	0.72 ± 0.10
Ramachandran plot statistics^c^	
Residues in most-favored regions (%)	72.6
Residues in additionally allowed regions (%)	26.2
Residues in generously allowed regions (%)	0.6
Residues in disallowed regions (%)	0.6

a α-helical dihedral angle restraints imposed for the backbone based on the CSI.

b Calculated for an ensemble of the 20 lowest-energy structures.

c Based on PROCHECK analysis (Laskowski et al., 1996).

The structure of the Nrd1p CID-Trf4p NIM complex shows that the specific recognition of the NIM is facilitated by a hydrophobic β turn in the C-terminal region and negatively charged residues in the N-terminal region of the peptide (Figure 4B). These two elements are conserved in budding yeast. Akin to the CTD, the NIM peptide adopts the β turn conformation at Asn580-Pro581-Tyr582-Thr583 (Figure 4C). Pro581 and Tyr582 of the NIM β turn, along with the preceding Tyr579, dock into a hydrophobic pocket of the Nrd1p CID that is formed by Ile29, Tyr67, Met126, Leu127, and Ile130 (Figures 4A and S4G). Tyr579 of the NIM also forms intramolecular stacking with Pro581, and the hydroxyl group of Tyr579 forms a hydrogen bond with a conserved Asp70 of Nrd1p CID (Figure 4A). Alanine substitution at position Tyr579 of the NIM strongly diminished the binding affinity for Nrd1p CID (Figures 4E and S5B), confirming the importance of the intricate interaction network of this residue. Consistently, the Asp70Ala variant of CID displayed a significant drop in binding affinity for the NIM peptide (Figures 4E and S5A). Tyr582 of the NIM β turn stacks with the methyl groups of Ile130 of the CID, and the perturbation of this interaction yields a 20-fold decrease in binding affinity (Ile130Lys variant).

The N-terminal region of NIM contains a stretch of negatively charged residues (Asp573-Asp574-Asp575-Glu576-Asp577), which contains the putative Ser5P mimic (Glu576) and interacts with a positively charged pocket formed by Lys21, Ser25, and Arg28 in the α1-α2 loop of CID (Figure 4B). In particular, the carboxyl groups of Asp577 and Asp575 of NIM form a hydrogen bond with Ser25 and Arg28 of the CID, respectively (these hydrogen bonds are inferred from the final ensemble of structures, and they are indirectly defined by neighboring proton-proton Nuclear Overhauser Effect [NOE]). Similarly, Asp573 of the NIM contacts Lys21 of the CID via a hydrogen bond between the carboxyl group and the side-chain amino group of Lys21. The importance of these contacts was further tested in a quantitative in vitro binding assay using FA. Aspartate (charge swapping) or arginine substitutions at positions Leu20, Lys21, Ser25, and Arg28 significantly decreased the binding affinity for the NIM peptide (Figures 4A, 4E, and S5A). The equivalent mutations in the NIM also showed a decrease in the binding affinity for Nrd1p CID, albeit with a lower magnitude. Notably, mutation of Glu576 to alanine or to arginine partially affected binding, indicating that the putative phosphomimic is important, but not essential, for the interaction (Figures 4A, 4E, and S5B). This suggests that the flanking aspartates of the Asp-rich stretch may substitute one another in the single-point mutants. Altogether, the structural and binding data show that Trf4p NIM is recognized by Nrd1p CID through two elements, hydrophobic β turn and Asp-rich stretch, which is a recognition mechanism similar to that used for the recognition of phosphorylated CTD.

### The Architecture of Interactions between the NNS Complex, the TRAMP, and the Nuclear Exosome

**Figure 5:**
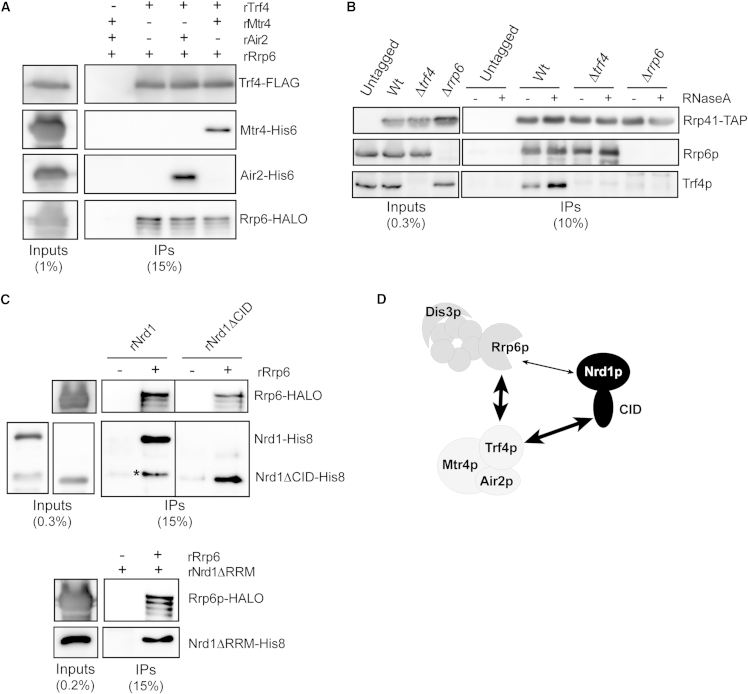
Analysis of the Interactions between the Exosome, the TRAMP, and Nrd1p (A) Pull-down experiments as in Figure 3B, using Trf4-FLAG as a bait and recombinant Rrp6-Halo, or His-tagged Air2 and Mtr4. The fraction of extract and immunoprecipitated material that is loaded on the gel is indicated. (B) Western blot analysis of factors associated with the core exosome. Rrp41p-TAP eluates purified from wild-type, *trf4Δ*, or *Δrrp6* cells (IPs) were probed with anti-Rrp6 (1:1,000 dilution) and anti-Trf4 antibodies, respectively. (C) Pull-down experiment using recombinant Rrp6-Halo as bait and His-tagged Nrd1 variants as indicated. An asterisk indicates a proteolytic fragment of rNrd1 that lacks most of the CID domain. (D) Schematic summarizing the protein-protein interactions identified in this work. A thinner arrow is used to indicate that the interaction between Rrp6p and Nrd1p cannot be detected in vivo.

Having established that Nrd1p interacts directly with Trf4p via the CID-NIM interaction, we undertook the characterization of the interactions linking the NNS complex and TRAMP with the nuclear exosome and Rrp6p. The strong and stable binding of TRAMP to the NNS complex (Figure 2A) contrasts with the weak, mostly RNA-dependent signal that we observed for Rrp6p and the core exosome in Nrd1p immunoprecipitates (Figure S3A). We considered the possibility that Rrp6p and the core exosome might be recruited to the NNS complex via the Nrd1p-TRAMP interaction. However, the molecular details of the interaction between TRAMP and the nuclear exosome/Rrp6p have not been elucidated. Therefore, we first performed pull-down assays using *E. coli* extracts expressing recombinant TRAMP components and halo-tagged Rrp6. RNase treatment was included to prevent detection of RNA-dependent interactions. These experiments revealed a clear and direct interaction between rTrf4 and rRrp6 irrespective of the presence of rMtr4 or rAir2 (Figure 5A). To assess whether TRAMP also interacts with the nuclear exosome independently of Rrp6p, we immunoprecipitated the core exosome using TAP-tagged Rrp41p from wild-type or *Δrrp6* cells. As shown in Figure 5B, we detected significant signals for Trf4p in purified core exosome from wild-type, but not from *Δrrp6* cells, strongly suggesting that Rrp6p bridges TRAMP and the core exosome.

The interaction we detected in vivo between Rrp6p and Nrd1p is strongly dependent on the presence of RNA (Figure S3A). However, we cannot completely exclude the existence of direct but weak or transient contacts in vivo that are not easily detected under our assay conditions. Therefore, we decided to assess whether a direct interaction could be detected between recombinant Rrp6 and Nrd1. To address the RNA dependency of the interaction, we either treated our extracts with RNase A or used a variant of rNrd1 lacking the RNA binding domain (rNrd1-ΔRRM). As shown in Figure 5C, we observed an interaction between rRrp6 and rNrd1 or rNrd1-ΔRRM. Importantly, this interaction was not mediated by the CID since it was also detected with rNrd1ΔCID. From these experiments, we conclude that Rrp6p interacts directly with Nrd1 in a CID-independent manner and with Trf4p. The latter interaction allows the association of TRAMP with the nuclear exosome (Figure 5D).

### Nrd1p Interaction with Trf4p Stimulates RNA Polyadenylation by TRAMP

**Figure 6:**
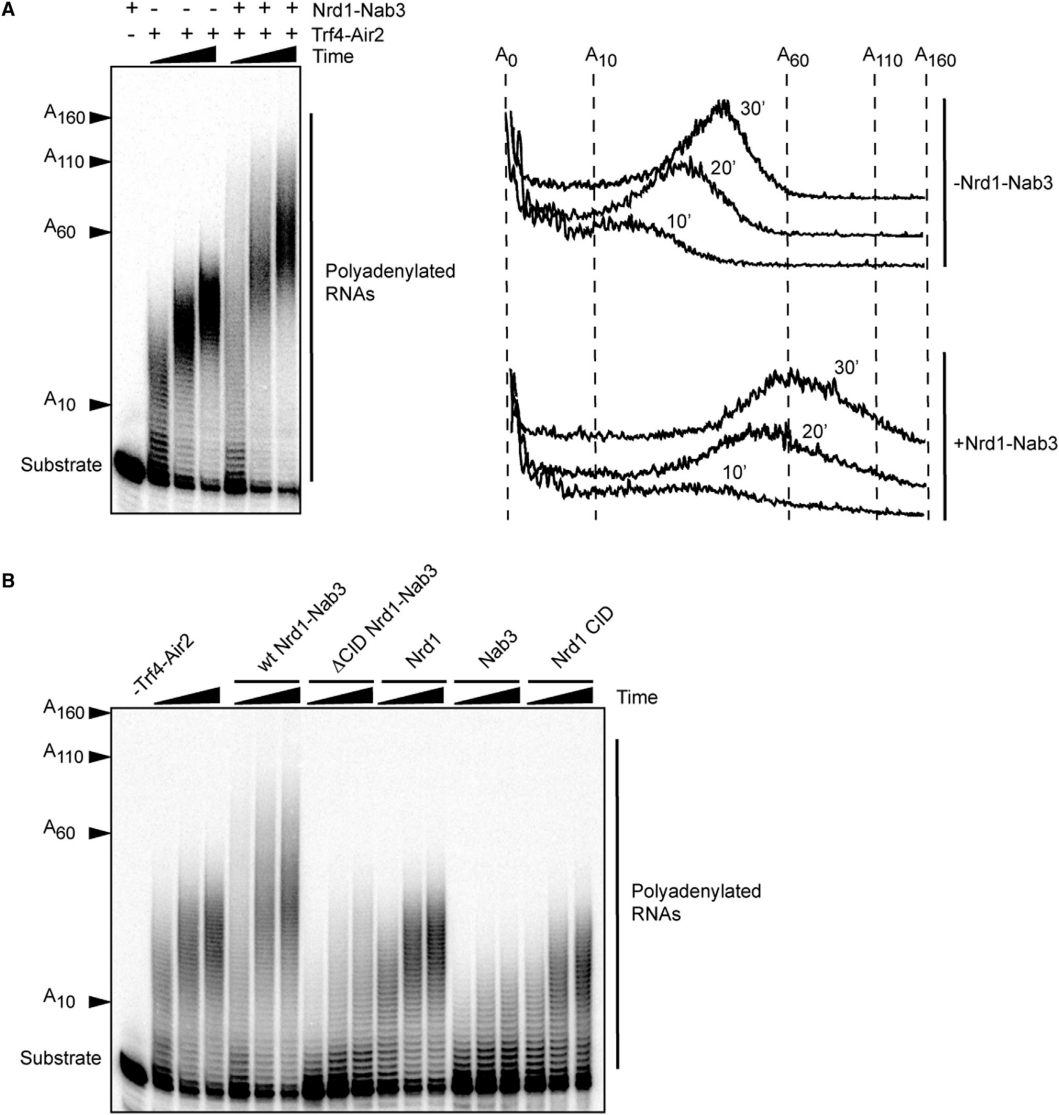
Analysis of the Effect of rNrd1-Nab3 on the Polyadenylation Activity of rTrf4-rAir2 In Vitro (A) Polyadenylation assays with recombinant Trf4-Air2 in the absence or in the presence of recombinant Nrd1-Nab3. Left: PAGE analysis of polyadenylated species at 10, 20, and 30 min reaction time. The position of the substrate and the number of added As is indicated. Right: lane scans of the gel shown on the left. (B) Polyadenylation assays as in (A) to individually assess the role of rNrd1, rNab3, and the Nrd1 CID domain in stimulating polyadenylation by rTrf4-rAir2. Reactions were performed with rTrf4-rAir2 in the presence of the indicated proteins or protein complex.

The Trf4-Air2 heterodimer possesses a distributive poly(A) polymerase activity in vitro (LaCava et al., 2005, Vanácová et al., 2005, Wyers et al., 2005). Considering the high affinity of Nrd1p and Nab3p for their RNA targets (Carroll et al., 2007, Hobor et al., 2011, Porrua et al., 2012), and the strong interaction of Nrd1p with Trf4p, we surmised that the Nrd1-Nab3 heterodimer might stimulate polyadenylation of NNS substrates by Trf4p-Air2p, for instance by improving recruitment or by stabilizing the Trf4-Air2 complex on the RNA. Therefore, we assessed the effect of adding rNrd1 and rNab3 to in vitro polyadenylation assays with recombinant Trf4p-Air2p (Figures 6 and S6A). We first used a 40-mer RNA substrate containing Nrd1p and Nab3p binding sites that we have previously shown to efficiently bind the Nrd1-Nab3 heterodimer in vitro and elicit efficient NNS-dependent transcription termination in vivo (Porrua et al., 2012). As shown in Figure 6A, using limiting concentrations of rTrf4-rAir2 relative to the substrate, polyadenylation was markedly stimulated by the addition of purified recombinant Nrd1p-Nab3p, resulting in a longer length of the added poly(A) tails. We did not observe any substantial increase in the fraction of RNA that is polyadenylated in response to the addition of rNrd1-Nab3 (compare the levels of nonadenylated substrate in Figures 6A and 6B), suggesting that, at least in vitro, stimulation preferentially occurs on molecules that have already undergone a polyadenylation cycle.

Stimulation was dependent on the interaction between rNrd1 and rTrf4 because it was abolished by the use of rNrd1ΔCID instead of wild-type rNrd1 (Figure 6B). However, the addition of recombinant CID alone did not enhance the polyadenylation activity of rTrf4-rAir2, ruling out that stimulation could result from an allosteric activation of Trf4p by the CID. The whole rNrd1-rNab3 heterodimer was required because neither subunit alone could enhance polyadenylation; rather, we observed a mild inhibition of rTrf4p-rAir2 activity when adding only rNab3 to the reaction (Figure 6B). Finally, high-affinity binding of rNrd1-Nab3 to the RNA was required because no significant stimulation could be obtained when using a mutant substrate that binds the heterodimer with an affinity ≈20-fold lower than that of the wild-type version (Figures S6B and S6C). Taken together, our results strongly suggest that the simultaneous interaction of Nrd1p-Nab3p with Trf4p-Air2p and the RNA enhances polyadenylation, most likely by favoring or stabilizing the association of Trf4p-Air2p with its substrate.

## Discussion

The Nrd1-Nab3-Sen1 complex is of major biological relevance because of its central role in the biogenesis of snRNAs and snoRNAs and in the control of pervasive transcription in connection with the exosome and TRAMP complexes (Schulz et al., 2013). The CID domain of Nrd1p has retained special attention because of its ability to bind the CTD of RNAPII. However, despite a number of biochemical and structural studies (Vasiljeva et al., 2008, Kubicek et al., 2012), its actual function in vivo has remained mysterious. In this work, we reexamine the role of the CID and demonstrate that it plays important roles in the efficiency of transcription termination and in RNA degradation.

### The Role of Nrd1p CID in Transcription Termination

The genome-wide occupancy of RNAPII upon deletion of the CID reported here clearly reveals a widespread role for this domain in transcription termination of CUTs, snoRNAs, and to some extent, small ORFs (Figures 1 and S1), which is consistent with the known landscape of NNS targets (Steinmetz et al., 2006b). While this work was in progress, Heo et al. (2013) reported similar findings using CID-swapped chimeric constructs. However, because deletion of the CID does not prevent NNS termination, the interaction between Nrd1p and the RNAPII CTD only impacts the efficiency of the process.

Deletion of the CID has a strong impact on Nrd1p recruitment, even in the presence (as in CUTs) of clusters of Nrd1 and Nab3 sites on the nascent RNA, which have been shown to be required for recruitment (Gudipati et al., 2008). This can be best explained if recruitment depends synergistically (and not redundantly) on the interaction of the Nrd1-Nab3 complex with both the CTD and the nascent RNA.

Termination of mRNA coding genes depends on the cleavage and polyadenylation factor/cleavage factor (CPF/CF) that also interacts with the nascent RNA and the CTD. We have previously shown that the CPF and the NNS complex have partially overlapping sequence requirements and that the same RNA sequence can be used as a terminator by either complex depending only on the distance from the transcriptional start (Gudipati et al., 2008, Porrua et al., 2012). It is likely that early recruitment of the NNS complex via the Nrd1p CID-CTD interaction kinetically favors the appropriate recognition of RNA binding sites that could otherwise be bound by the CPF complex, impairing termination by the NNS pathway.

### The CID Mediates the Connection between the NNS Complex and TRAMP

Our results strongly suggest that the function of the CID in RNA degradation relies on the interaction with a CTD-like motif in Trf4p. The strong association between the termination complex and the corresponding poly(A) polymerase mirrors the association of Pap1p with the CPF complex, suggesting that in both cases the appropriate poly(A) polymerase is brought in the proximity of the 3′-OH of the newly released (or cleaved) RNA, thus preventing or limiting spurious cross-processing events.

The two alternative forms of TRAMP containing either Trf4p or Trf5p have partially redundant functions, but the former predominates in the degradation of CUTs and the processing of snRNAs and snoRNAs (San Paolo et al., 2009). Because Trf5p does not contain a NIM (Figure S3D), this different target specificity can now also be explained by the interaction of the NNS complex with Trf4p.

In contrast to the robust interaction of the NNS complex with TRAMP, we found the interaction of Rrp6p and the exosome with the NNS complex in vivo to be strongly RNA dependent (Figure S3A), which is in apparent discrepancy with a previous report (Vasiljeva and Buratowski, 2006). Nevertheless, we could show a direct interaction between Rrp6p and Nrd1p in vitro that is independent from the CID, suggesting that this interaction is too weak to withstand our immunoprecipitation conditions or that it forms only transiently in vivo.

### Functional Significance of the NNS-TRAMP Interaction in Degradation

It is known that the RNA quality control factors target a vast repertoire of defective molecules that are sorted because of kinetic competition between RNA degradation and processing (Bousquet-Antonelli et al., 2000, Gudipati et al., 2012). However, when alternative routes to discarding are not desired, it is crucial to enforce degradation by the use of specific adaptors.

The NNS complex fulfils such a function by recruiting the TRAMP to its targets after transcription termination via the CID-NIM interaction. This could stimulate degradation because of enhanced polyadenylation of the substrates, but it is also possible that degradation is stimulated by a poly(A)-independent mechanism (Callahan and Butler, 2010, LaCava et al., 2005, Wyers et al., 2005), maybe by recruiting the exosome by virtue of the direct Trf4p-Rrp6p interaction (Figure 5).

We note that stimulation preferentially occurs on a fraction of already polyadenylated RNAs rather than on the nonadenylated substrate. It is possible that Nrd1p-Nab3p and Trf4p-Air2p compete for binding to the short RNA substrates we used in our assays, which is also suggested by the inhibition of the polyadenylation reaction when rNab3 alone or rNrd1ΔCID-Nab3 are used (Figure 6B). The emergence of a poly(A) tail likely provides a binding platform in the vicinity of the substrate 3′-OH that would be preferentially bound by Trf4p-Air2p.

It has previously been suggested that the RNA helicase Mtr4p modulates the activity of Trf4p-Air2p, restricting the addition of poly(A) tails after 3–4 nt in vitro (Jia et al., 2011). Although very short poly(A) tails (≈4 nt) have also been observed in vivo (Jia et al., 2011, Wlotzka et al., 2011), it must be noticed that these represent average steady-state lengths, resulting from an equilibrium between synthesis and degradation. When degradation is impaired, Trf4p-dependent poly(A) tails in vivo are longer (Wyers et al., 2005; D.L., unpublished data), most likely within the range required to allow threading of substrates through the exosome channel (i.e., roughly 30 nt). The antagonistic impact of the Nrd1-Nab3 complex and Mtr4p on Trf4p activity might imply a temporal regulation of polyadenylation, restricted by Mtr4p early after transcription to prevent the binding of Pab1p (Jia et al., 2011) and stimulated later on by Nrd1p-Nab3p to favor degradation. Alternatively, Mtr4p and Nrd1p-Nab3p might modulate Trf4p activity at different substrates.

### Recognition of Hydrophobic β Turn Hairpin and Electronegative Stretch by the CID

The CTD and NIM share a sequence element that can form a β turn (Figure 4). The binding mode of the NIM peptide at the β turn conformation resembles other previously determined structures of CTD bound to CIDs of Pcf11p, SCAF8, Rtt103p, and Nrd1p (Figures 4C and S7) (Meinhart and Cramer, 2004, Becker et al., 2008, Lunde et al., 2010, Kubicek et al., 2012). However, in contrast to these CID-CTD complexes, the β turn of the NIM peptide has more extensive hydrophobic contacts with Nrd1p due to the presence of Tyr582, the third residue of the β turn not present in any of the CTD repeats (Jasnovidova and Stefl, 2013). The stacking of Tyr582 with Ile130 of Nrd1p significantly contributes to the overall increase of binding affinity of NIM to Nrd1p in comparison to the phosphorylated CTD. The other region of the NIM peptide that contributes to the overall affinity is the aspartate-rich region located at the N terminus that intimately interacts with the electronegative pocket of Nrd1p (Figure 4B). To some extent, this mimics the recognition of phosphorylated CTD, but it involves more contacts, strengthening the overall binding affinity (Figure 4C). It is also likely that other proteins contain these two elements with the same arrangement and therefore could interact with CID-containing proteins in a similar manner.

### A Role for the CID in Coordinating Transcription Termination and RNA Degradation

Our structural data together with our competition assays demonstrate that the interactions of the CID with the CTD and the NIM are mutually exclusive (Figures 4 and 5). This implies the existence of at least two distinct forms of the NNS complex, one associated with the polymerase and the other associated with the TRAMP, which could represent pre- and posttermination forms of the NNS complex. Although we show that the affinity of the CID for the NIM is 100-fold higher than that for the CTD, the real balance between the two alternative complexes also depends on the number of RNAPII and Trf4p molecules available for interaction and, importantly, on the number of interaction targets, which is presumably higher for RNAPII (25 possible diheptapeptides in the CTD).

**Figure 7:**
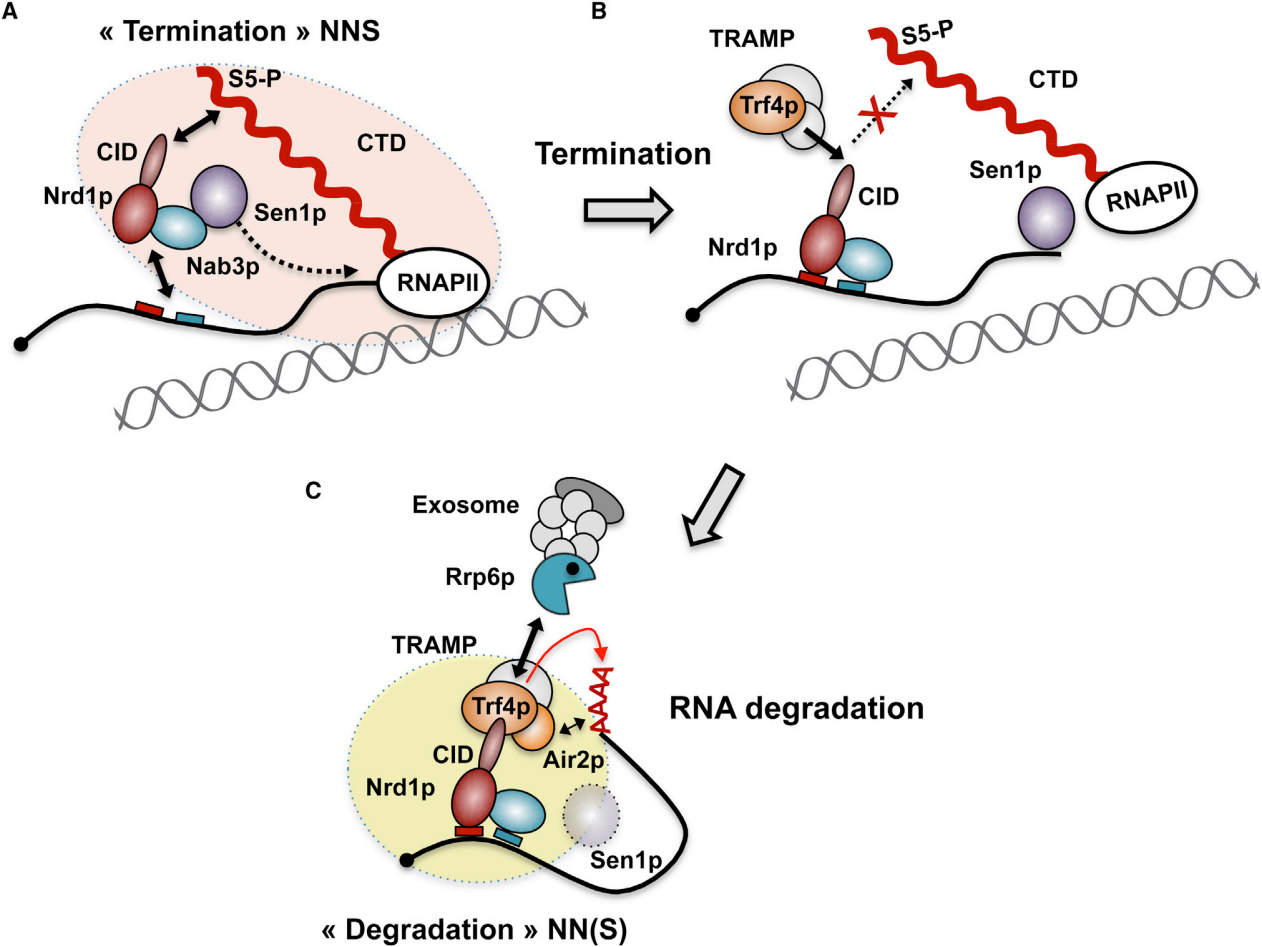
Model for the Coordination of Transcription Termination with RNA Degradation at NNS Targets (A) The NNS complex interacts with the Ser5P form of the CTD via the CID of Nrd1p and with the nascent RNA (boxes indicate recognition motifs for the Nrd1-Nab3 heterodimer), which defines a termination form of the complex (orange shaded). (B) Concomitantly with or subsequently to dissociation of the elongation complex by Sen1p, Trf4p interacts with the CID of Nrd1p, replacing the CID-CTD interaction and allowing the release of Nrd1p-Nab3p from RNAPII. (C) Polyadenylation of the transcript by TRAMP (in the presence or absence of Mtr4p) is stimulated by the simultaneous interaction of Nrd1p-Nab3p with the RNA and with Trf4p. Trf4p also might recruit the exosome via the interaction with Rrp6p, possibly favoring subsequent degradation of the transcript in a polyadenylation- independent manner. The alternative interaction of Nrd1p with Trf4p (instead of the CTD) defines a degradation form of the NNS complex (yellow shaded) that might or might not contain Sen1p.

We propose that by virtue of its alternative interactions, the CID controls the handover of the NNS complex from RNAPII to the TRAMP, which would temporally coordinate the two functions of the complex. Regulation of NNS and TRAMP function might be reciprocal because interaction with the TRAMP might control the release of Nrd1p from RNAPII (Figure 7). This could be important for the downstream function of the NNS complex in processing/degradation, but also for making the complex available for the interaction with new elongating RNAPIIs.

Our results open up the interesting possibility that the dynamics of factors interacting with the CTD throughout the transcription cycle is regulated not only by the enzymes responsible for CTD modifications and proline isomerizations, but also by competitive interactions with proteins containing CTD-like motifs.

## Experimental Procedures

### Yeast Strains, Plasmids, and Standard Analyses

ChIP and ChIP-chip experiments were performed as previously described (Mayer et al., 2010, Rougemaille et al., 2008). More complete details for these experimental procedures as well as construction of plasmids, yeast strains, RNA analyses, and standard biochemical analyses can be found in the Supplemental Experimental Procedures.

### ChIP-Chip Occupancy Profiling

ChIP-chip data analysis was performed essentially as described (Mayer et al., 2010). Briefly, we first performed quantile normalization between replicate measurements and averaged the signal for each probe over the replicate intensities. ChIP enrichments were obtained by dividing ChIP intensities by the corresponding input intensities. The normalized ChIP signal at each nucleotide was calculated as the median signal for all probes overlapping this position. Profiles were smoothed using running median smoothing with a window half size of 75 bp. To average profiles over feature classes, features were aligned at their transcription start sites (TSSs) and transcription termination sites (TTSs) or poly(A) addition sites for protein-coding genes, scaled to the same length (i.e., the median length of all transcripts in the class), and averaged by calculating the 5% trimmed mean at each genomic position. For better comparison of CUTs and small-sized protein-coding genes, features of both classes with a length between 350 and 550 bp were selected and scaled to a length of 450 bp. Note that annotation of CUTs does not generally take into account the 3′ end heterogeneity that is characteristic of these transcripts. Medium-sized protein-coding genes were selected by taking the 50% most highly expressed genes (Dengl et al., 2009) that were at least 200 bp away from neighboring genes, with an ORF length of 1,238 ± 300 bp.

Note that because the size of CUTs and snoRNAs is generally similar to or lower than the resolution of the ChIP technique (200–300 nt), the increased downstream RNAPII signal also bleeds over the body of the metagene. The apparent RNAPII increase within CUTs is also due to the fact that these genes have multiple termination sites (Neil et al., 2009, Wyers et al., 2005) that fall within the coordinates of each annotation (and therefore of the metagene). The possibility that deletion of the CID generally affects transcription initiation at CUTs and snoRNAs is very unlikely because (i) the RNAPII increase in *nrd1ΔCID* relative to the WT is always minimal at the 5′ end and progressively increases toward the termination region (Figures 1A–1C and 1E-F) and (ii) we did not detect a significant increase in the levels of mature snRNAs (data not shown).

### NMR Spectroscopy

All NMR spectra for the backbone and side-chain assignments of 0.5–2.0 mM uniformly ^15^N,^13^C-labeled Nrd1p CID in 50 mM sodium phosphate buffer (pH 8.0), 100 mM NaCl (90% H_2_O/10% D_2_O) were recorded on Bruker AVANCE 700 and 950 MHz spectrometers equipped with a cryoprobe at a sample temperature of 20°C. The spectra were processed using NMRPipe package (Delaglio et al., 1995), and the protein resonances were assigned manually using Sparky software (T.G. Goddard and D.G. Kellner, University of California, San Francisco). The ^1^H, ^13^C, and ^15^N chemical shifts of the bound form of Nrd1p CID were assigned as described previously (Kubíček et al., 2011, Kubicek et al., 2012). All distance constraints were derived from the 3D ^15^N- and ^13^C-separated NOESYs and 2D ^1^H-^1^H NOESY (with mixing time of 80 ms) collected on a 950 MHz spectrometer. Intermolecular distance constraints were obtained from the 3D F_1_-^13^C/^15^N-filtered NOESY-[^13^C,^1^H]-HSQC experiment (Peterson et al., 2004, Zwahlen et al., 1997), with a mixing time of 150 ms on a 950 MHz spectrometer. Intramolecular distance constraints of the bound Trf4p NIM peptide (unlabeled) were derived from a 2D F_1_, F_2_-^13^C/^15^N-filtered [^1^H,^1^H]-NOESY (t_m_ = 150 ms) (Peterson et al., 2004, Zwahlen et al., 1997). The NOEs were semiquantitatively classified based on their intensities in the 2D and 3D NOESY spectra. The structure determination was performed as described previously (Kubicek et al., 2012).

### Polyadenylation Assays

Polyadenylation reactions were performed at 30°C in a final volume of 20 μl containing 2 nM 5′ end-labeled RNA substrate and 1 nM recombinant Trf4-Air2 in 20 mM Tris-HCl (pH 7.5), 100 mM NaCl, 0.5 mM MgCl_2_, 10% glycerol, 0.01% nonidet P-40, and 1 mM dithiothreitol in the presence of RNase inhibitors. Reactions were started upon addition of 2 μl of an ATP-MgCl_2_ mixture (20 mM each) and stopped at different time points by collecting 4 μl aliquots and mixing them with an equal volume of loading buffer (80% formamide, 0.05% w/v bromophenol blue, and 0.05% w/v xylen cyanol). RNAs were denatured for 5 min at 75°C and separated by 10% (w/v) denaturing PAGE. After electrophoresis, gels were dried and analyzed using a Phosphorimager scanner (GE Healthcare). To assess the effect of rNrd1 and rNab3 on the polyadenylation activity of rTrf4-Air2, individual proteins or the heterodimeric complex were added to the reaction at a 3 nM final concentration and incubated for 10 min at 30°C before starting the reaction.

## Author Contributions

A.T. and O.P. designed and performed molecular biology, genetic, and biochemistry experiments. T.K. performed biochemical and structural analyses. M.L. performed genome-wide ChIP-chip experiments and analyzed the data. K.K. carried out NMR experiments and contributed to NMR data analyses. A.F. performed ChIP experiments. F.L. constructed strains. S.V. designed experiments. P.C. designed experiments. R.S. designed experiments and contributed to structural analyses. D.L. designed experiments and analyzed the data. All authors discussed the results. R.S. directed the research for all the studies performed at Masaryk University, notably the determination of the solution structure and part of the biochemical analyses. O.P. directed part of the research performed in the Centre de Génétique Moléculaire. Specifically, she directed the work of A.T. and designed experimental strategies relating to the discovery and functional characterization of the NIM. D.L. partially directed the work performed in the Centre de Génétique Moléculaire, contributing to the definition of the general experimental strategies and to the coordination of the research performed in the two main sites. D.L. also directed the work on the genome-wide analyses of Nrd1ΔCID and RNAPII distribution. O.P., R.S., and D.L. wrote the manuscript.

## Accession Numbers

The genome-wide ChIP-chip data are accessible with the number E-MTAB-2175.

The atomic coordinates and restraints for the Nrd1p CID-Trf4p NIM complex have been deposited in the Protein Data Bank under ID code 2MOW.

## Supplemental Information

Document S1. Supplemental Experimental Procedures and Figures S1–S7

Document S2. Article plus Supplemental Information
