# Supplementary material for: Molecular Basis for Coordinating Transcription Termination with Noncoding RNA Degradation
Source: Mol Cell. 2014 Aug 7;55(3):467–81. doi: 10.1016/j.molcel.2014.05.031 (PMC4186968; doi:10.1016/j.molcel.2014.05.031)
Supplement: Document S1. Supplemental Experimental Procedures and Figures S1–S7 [file mmc1.pdf]

Molecular Cell, Volume *55*

Supplemental Information

Molecular Basis for Coordinating Transcription Termination with Noncoding RNA Degradation

Agnieszka Tudek, Odil Porrua, Tomasz Kabzinski, Michael Lidschreiber, Karel Kubicek, Andrea Fortova, François Lacroute, Stepanka Vanacova, Patrick Cramer, Richard Stefl, and Domenico Libri

Supplemental figures

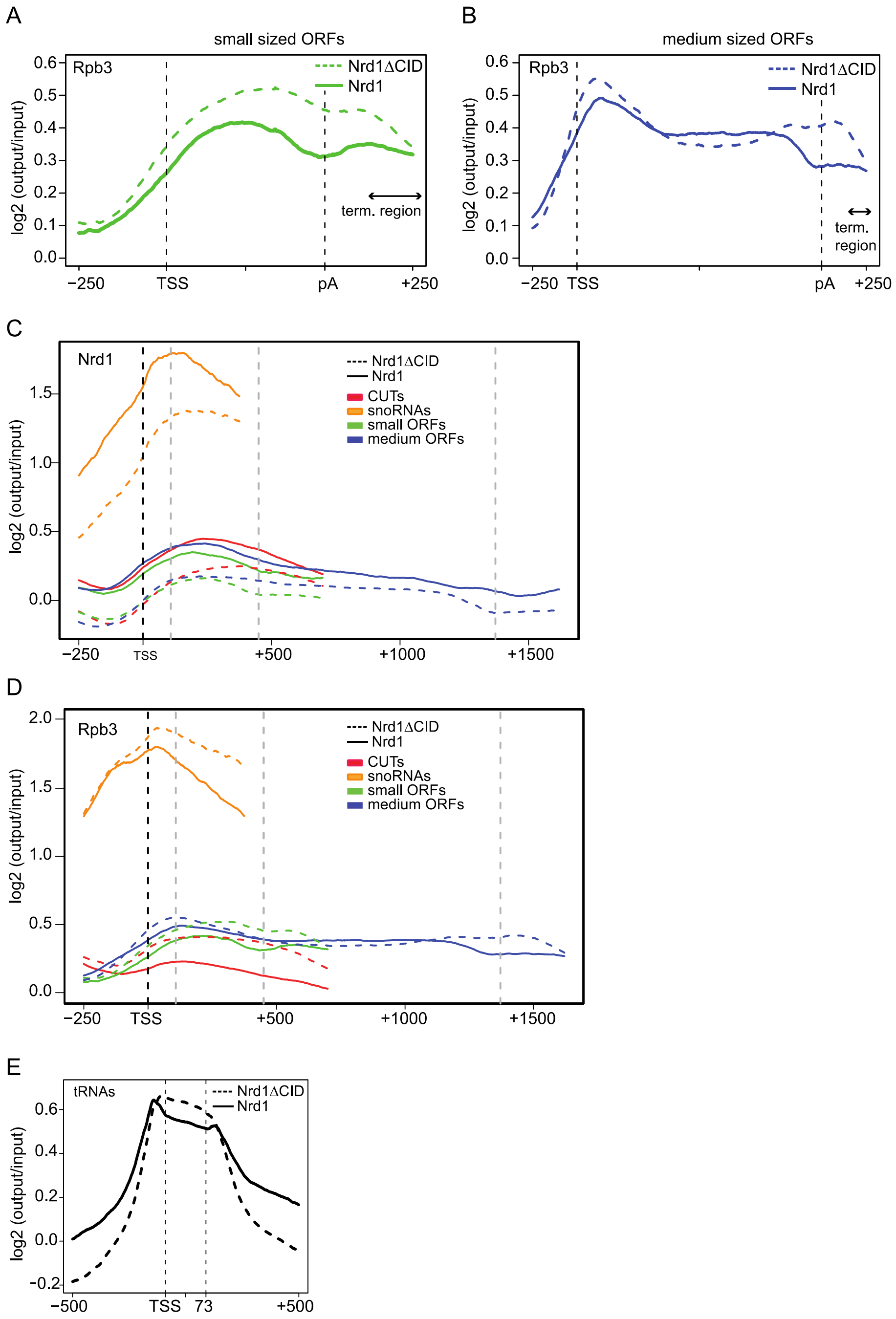

Figure S1: Related to figure 1. Metagene analysis of RNAPII distribution for *NRD1* and *nrd1ΔCID* strains at ORFs of the same size as CUTs (400-500nt, A) or larger (900-1500nt, B). All features have been scaled and aligned to the transcriptional start (TSS) and the poly(A) sites (pA), indicated by grey dotted lines. The approximate region of termination is indicated. **C**. Metagene analysis of distribution of Nrd1p occupancy as in figure 1G, with no normalization to RNAPII (Rpb3) signals. **D.** Metagene analysis of Rpb3p occupancy for the different classes of features as in figures 1B-F but using the same scale. **E**. Metagene analysis of Nrd1p and Nrd1ΔCIDp distribution at tRNA genes. tRNA genes have been aligned to their TSS. The median length of the distribution (73) is also indicated (grey dotted lines).

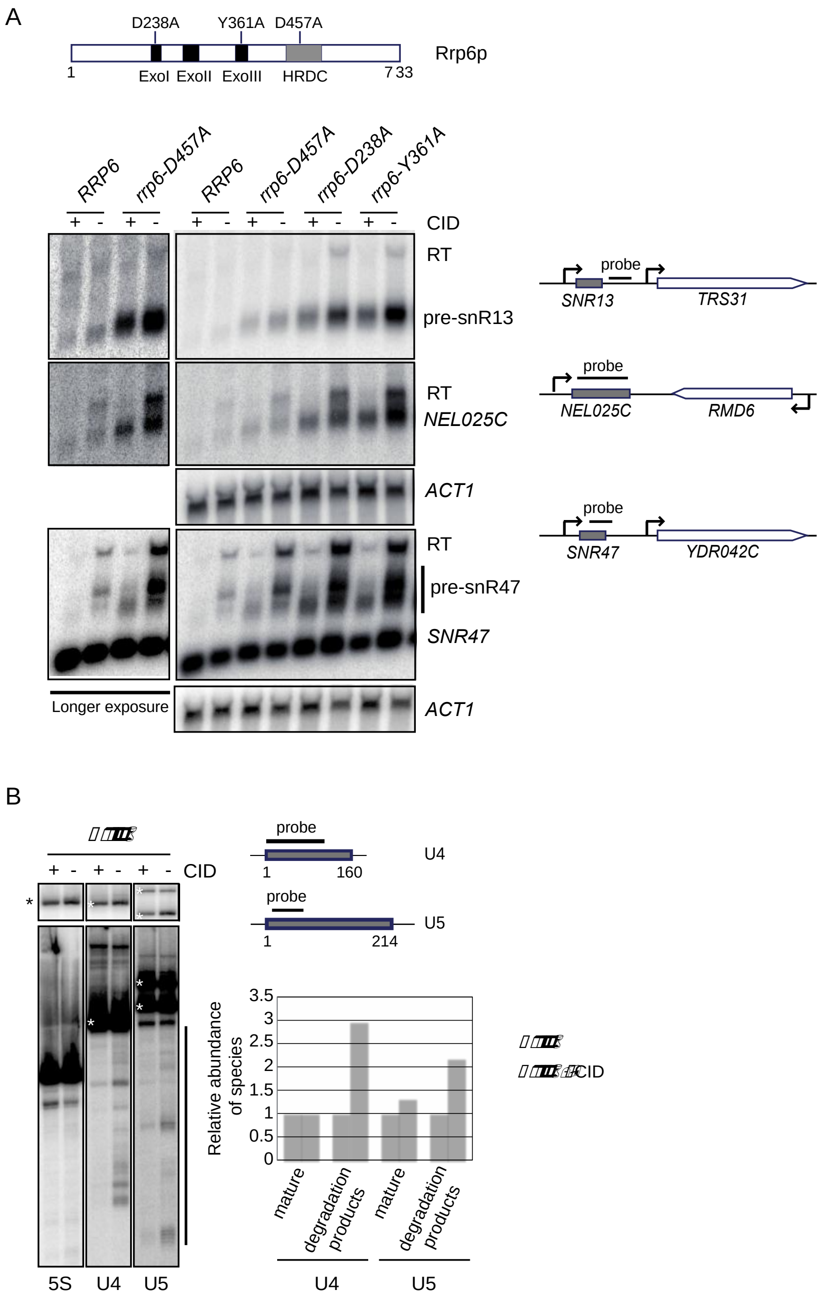

Figure S2: Related to figure 2. Effects of CID deletion on the steady state levels of several classes of RNAs. **A**. Northern blot analyses of the *NEL025C* CUT, pre-snR13 and pre-snR47 in the presence or absence of the CID in different genetic backgrounds as indicated. The different *RRP6* mutants contain modifications in either the exonuclease (exoI-III) or the helicase RNase D C-terminal (HRDC) domains that impair degradation/processing of several RNAs *in vivo* (Phillips and Butler, 2003). The position of the read-through species observed in the absence of the CID is indicated (RT). The left panels are a longer exposure of the right panels to visualize the *NEL025C* and pre-snR13 transcripts that are unstable and poorly detectable in these strains. **B.** Northern blot analyses of the degradation products of U4 and U5 snRNAs (indicated by a black bar) that are stabilized in a Δ*rrp6*, *nrd1*Δ*CID* relative to a Δ*rrp6* strain. The 5S rRNA is shown as a control. The upper panels show short exposures of the same blots and the mature species are indicated by asterisks. The graph represents the abundance of the different species in the Δ*rrp6*, *nrd1*Δ*CID* strain relative to the Δ*rrp6* (set as 1), normalized by the 5S signal.

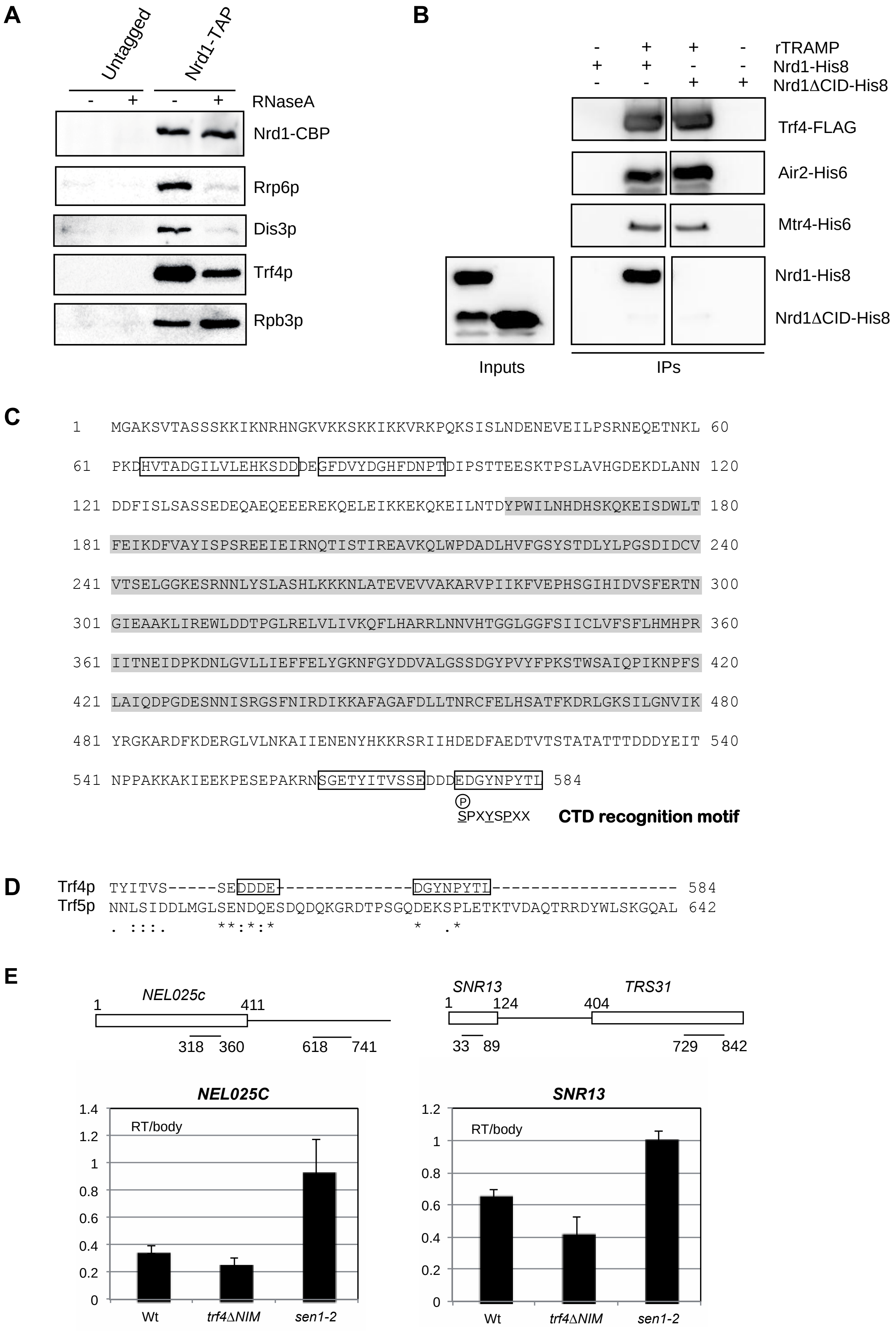
A
Untagged
Nrd1-TAP
- + - + RNaseA
Nrd1-CBP
Rrp6p
Dis3p
Trf4p
Rpb3p
B
- + + - rTRAMP
+ + - - Nrd1-His8
- - + + Nrd1ΔCID-His8
Trf4-FLAG
Air2-His6
Mtr4-His6
Nrd1-His8
Nrd1ΔCID-His8
Inputs
IPs
C
1 MGAKSVTASSSKKIKNRHNGKVKKSKKIKKVRKPQKSISLNDENEVEILPSRNEQETNKL 60
61 PKDHVTADGILVLEHKSDDDEGFDVYDGHFDNPTDIPSTTEESKTPSLAVHGDEKDLANN 120
121 DDFISLSASSEDEQAEQEEEREKQELEIKKEKQKEILNTDYPWILNHDHSKQKEISDWLT 180
181 FEIKDFVAYISPSREEIEIRNQTISTIREAVKQLWPDADLHVFGSYSTDLYLPGSDIDCV 240
241 VTSELGGKESRNNLYSLASHLKKKNLATEVEVVAKARVPIIKFVEPHSGIHIDVSFERTN 300
301 GIEAAKLIREWLDDTPGLRELVLIVKQFLHARRLNNVHTGGLGGFSIICLVFSFLHMHPR 360
361 IITNEIDPKDNLGVLLIEFFELYGKNFGYDDVALGSSDGYPVYFPKSTWSAIQPIKNPFS 420
421 LAIQDPGDESNNISRGSFNIRDIKKAFAGAFDLLTNRCFELHSATFKDRLGKSILGNVIK 480
481 YRGKARDFKDERGLVLNKAIIENENYHKKRSRIIHDEDFAEDTVTSTATATTTDDDYEIT 540
541 NPPAKKAKIEEKPESEPAKRNSGETYITVSSEDDDEDGYNPYTI 584
P
SPXYSPXX CTD recognition motif
D
Trf4p TYITVS-----SEDDDE--------------DGYNPYTI------------------- 584
Trf5p NNLSIDDLMGLSENDQESDQDQKGRDTPSGQDEKSPLETKTVDAQTRRDYWLSKGQAL 642
. :::. **:*:* * .*
E
NEL025c
1 411
318 360 618 741
SNR13 TRS31
1 124 404
33 89 729 842
NEL025C
RT/body
1.4 1.2 1 0.8 0.6 0.4 0.2 0
Wt trf4ΔNIM sen1-2
SNR13
RT/body
1.2 1 0.8 0.6 0.4 0.2 0
Wt trf4ΔNIM sen1-2

Figure S3: Related to figure 2. A. The interaction between Nrd1p and Rrp6-exosome is strongly RNA-dependent. Coimmunoprecipitation experiments with yeast extracts using Nrd1-TAP as bait. The presence of Rrp6p and the exosome in immunoprecipitates was assessed by western blot with anti-Rrp6p (1:1000 dilution, gift of T.H. Jensen) and anti-Dis3 (1:1000 dilution, gift of D. Tollervey). Trf4p and Rpb3p were detected as controls for direct protein-protein interactions using anti-Trf4 (1:1000 dilution) and anti-Rpb3 (1:2000, Neoclone). Nrd1p was revealed with an antibody against the CBP portion of the tag (1:5000, Millipore). **B.** Nrd1-TRAMP interaction depends on the presence of the CID. Pull-down experiments with bacterial protein extracts containing TRAMP subunits and either wt or ∆CID Nrd1p, using Trf4-FLAG as the bait. The different proteins were detected using either an anti-FLAG (1:3000 dilution, Sigma) or anti-polyhistidine (1:2000, SIGMA) antibodies. **C.** Analysis of Trf4p sequence with ANCHOR. The region that folds to form globular domains is shaded. Motifs contained in the N-terminal and C-terminal unstructured regions that scored high as putative protein-protein interaction sequences are indicated by open boxes. **D.** Alignment of *S. cerevisiae* Trf4p and Trf5p C-terminal regions using ClustalW2. Asterisks denote identical positions, colons and dots indicate amino acids with strong and weak similarity, respectively. Trf4p NIM is shown by an open box. **E.** Nrd1-Trf4 interaction via CID and NIM domains is not required for transcription termination. Transcription termination at *SNR13* and *NEL025c* was assessed by RNAPII ChIP-RT-PCR in wild type (wt) or *trf4∆NIM* strain. A temperature sensitive mutant of Sen1 (*sen1-2*) was used as a control for strain defective in the RNAPII termination of ncRNAs. The termination efficiency is determined as the occupancy of RNAPII downstream of the termination site (RT=read-through) relative to RNAPII in the body of the individual ncRNA. The scheme above the graphs shows the regions amplified in the RT-PCR. The experiment was performed in three independent biological experiments and the error bars display the standard deviation.

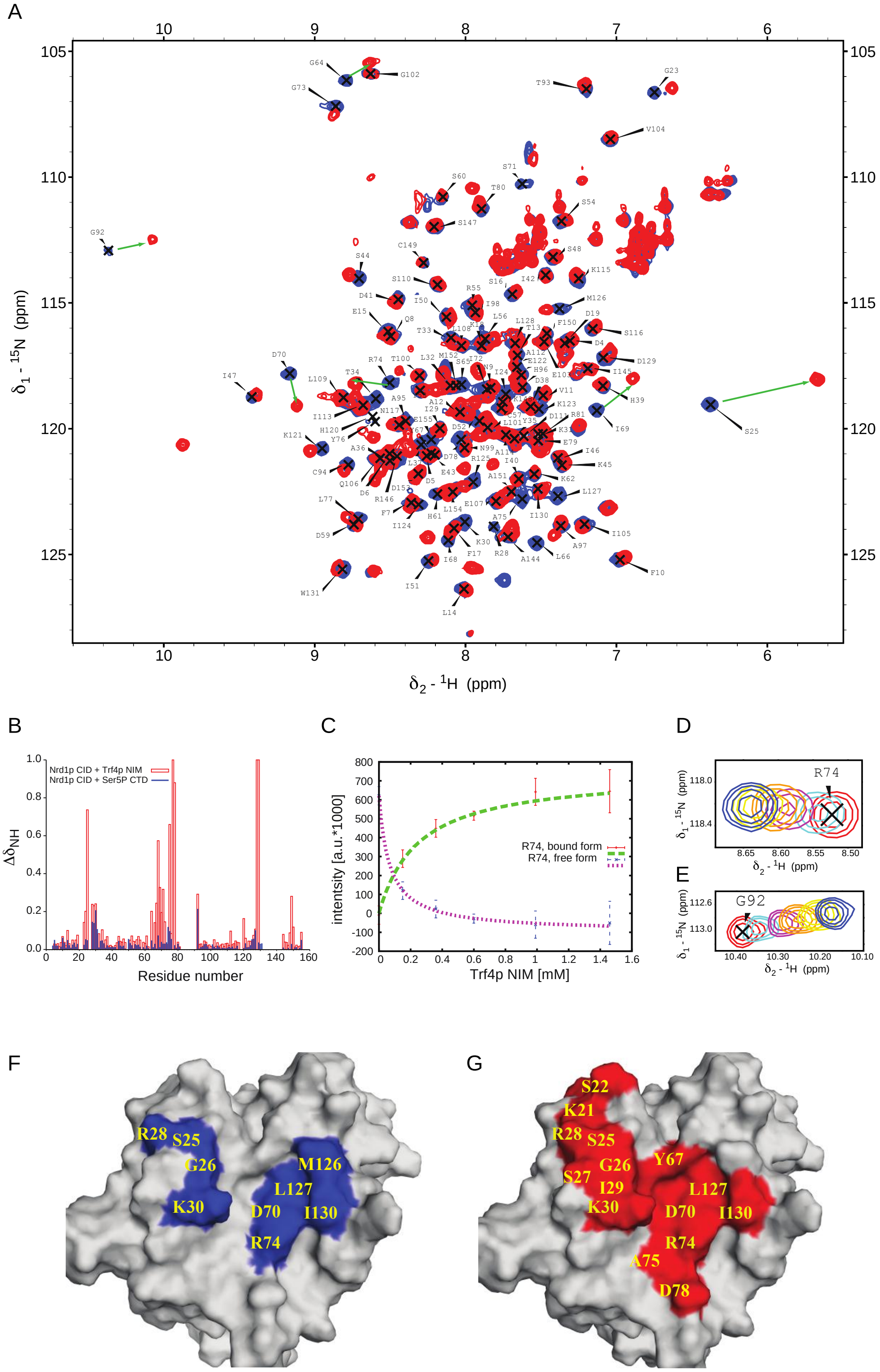
A
δ2 - 1H (ppm)
δ1 - 15N (ppm)
B
Nrd1p CID + Trf4p NIM
Nrd1p CID + Ser5P CTD
ΔδNH
Residue number
C
intensity [a.u.*1000]
Trf4p NIM [mM]
R74, bound form
R74, free form
D
R74
δ1 - 15N (ppm)
δ2 - 1H (ppm)
E
G92
δ1 - 15N (ppm)
δ2 - 1H (ppm)
F
R28
S25
G26
K30
M126
L127
D70
I130
R74
G
S22
K21
R28
S25
Y67
S27
G26
L127
I29
K30
D70
I130
R74
A75
D78

Figure S4: Related to figure 4. NMR data showing the interaction of Trf4p NIM with Nrd1p CID. **A**. NMR titration experiment of Nrd1p CID with Trf4p NIM. Overlays of ^{1}H-^{15}N HSQC spectra show the effect of addition of the unlabeled Trf4p NIM to ^{15}N-labeled Nrd1p CID at 20 °C. ^{1}H-^{15}N HSQC spectra of Nrd1p CID alone (in blue) and in complex with Trf4p NIM (ratio 1:1; in red) are shown. **B**. Quantification of chemical shift perturbations of Nrd1p CID upon binding to the NIM peptide (in red) and Ser5P CTD (in blue). The combined chemical shift perturbations are plotted versus the amino-acid residue number. **C**. Slow exchange regime in the Nrd1p CID–Trf4p NIM titration experiments. A plot of the amide peak intensity for residue R74 in the free and bound forms versus the NIM peptide concentration. **D**. Fast exchange regime in the Nrd1p CID–Ser5P CTD titration experiments. A close up for residue R74 in the ^{1}H-^{15}N HSQC spectra of Nrd1 CID alone (in red) and at the CTD peptide to protein ratios of 1/6 (in cyan), 2/6 (in magenta), 3/6 (in orange), 4/6 (in green), 5/6 (in yellow), 6/6 (in blue) are shown. **E**. The same as D. but for residue G92. F. Surface mapping of the chemical shift changes in the Nrd1p CID upon binding to the CTD. **G.** The same as **F**. but for the NIM binding surface.

A

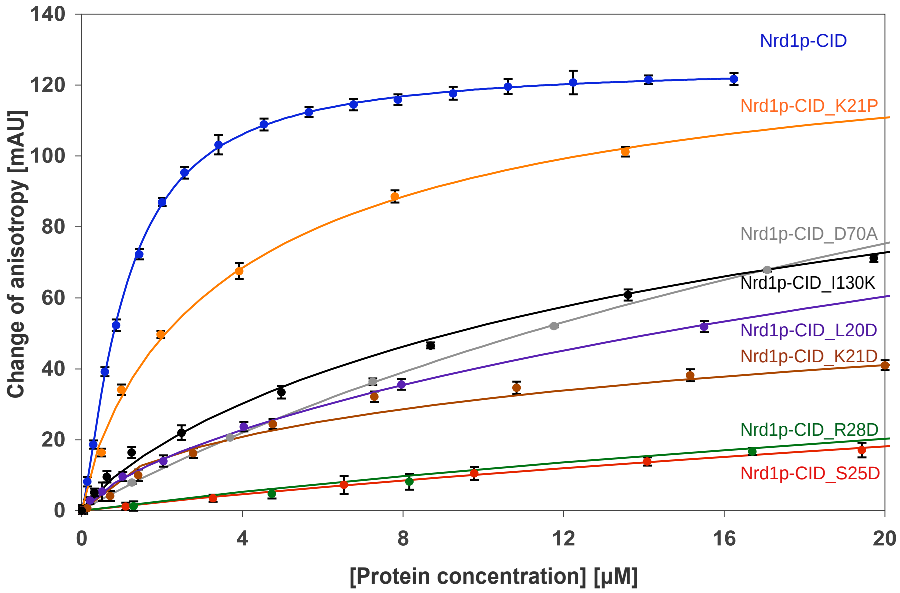

B

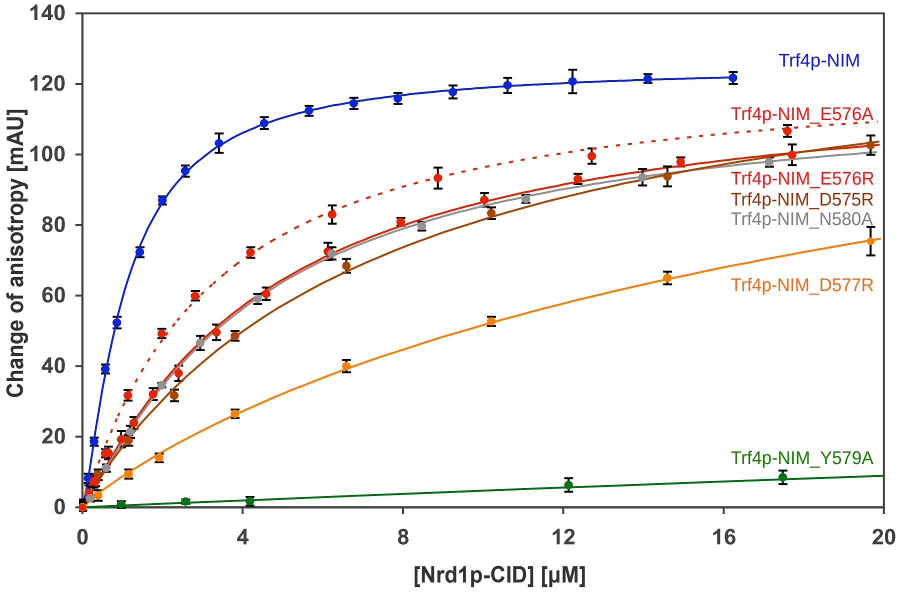

Figure S5: Related to figure 4. Equilibrium binding of Nrd1p CID to Trf4p NIM monitored by fluorescence anisotropy. **A**. Binding isotherms are shown for the wild type and mutants of Nrd1p CID to the NIM peptide. **B**. Binding isotherms are shown for Nrd1p CID to the wild type and mutants of Trf4p NIM.

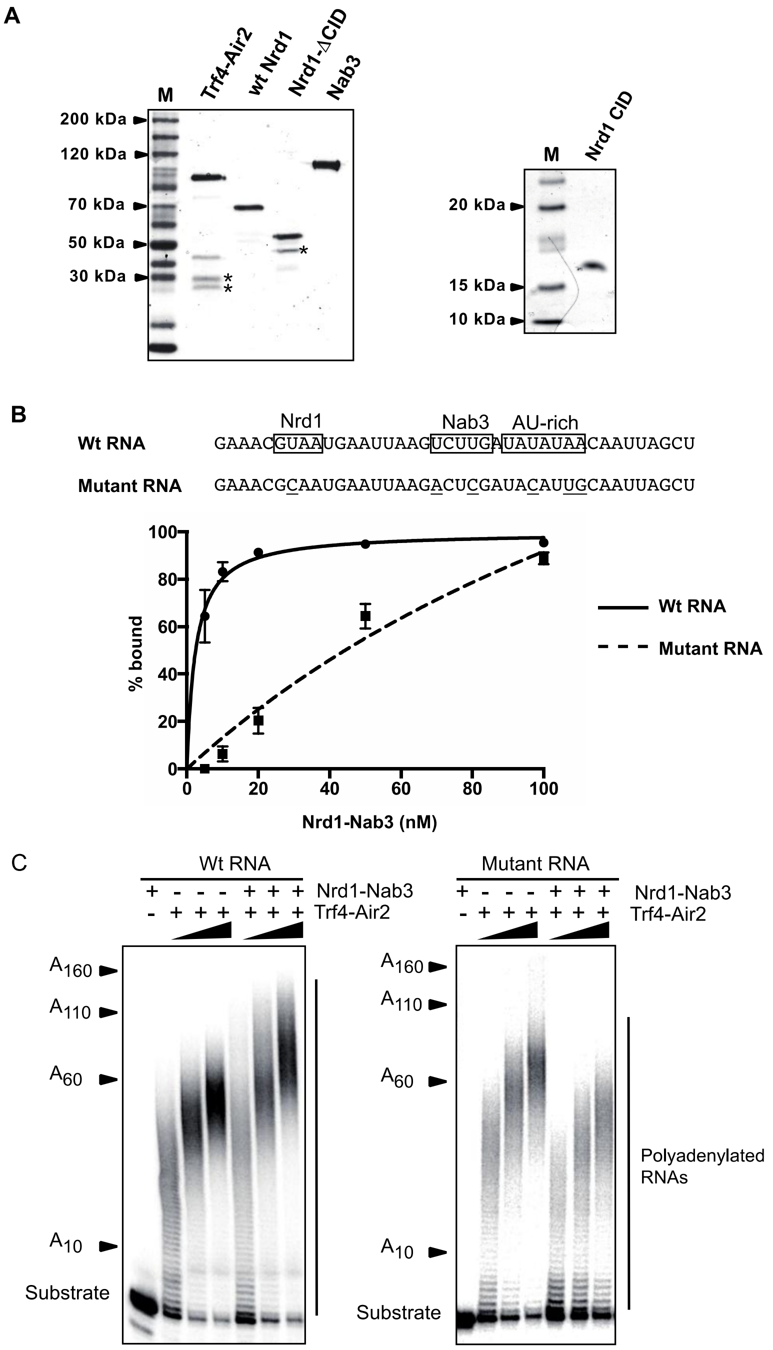

Figure S6: Related to figure 6. A. Coomassie stained SDS-PAGE showing 10 pmol of each protein preparation used (50 pmol of the CID). Asterisks indicate proteolytic fragments of Air2p (deletions at the C-terminal unstructured region) and Nrd1-ΔCIDp (N-terminal truncation) that constitutes approximately 20% of the full-length protein. M: molecular weight marker. **B.** Analysis of Nrd1p-Nab3p binding to either the wt RNA, containing motifs required for high-affinity RNA-binding (open boxes), or a mutant variant harboring point mutations designed to disrupt all motifs (mutations underlined). The plots correspond to affinity curves generated by the Prism software from values derived from three independent electromobility shift assays (see supplemental experimental procedures). Bars denote standard deviations. The affinity of Nrd1p-Nab3p for the wt RNA is 2.4 ± 0.3 nM. The affinity for the mutant RNA could not be accurately determined due to the poor fit of the curve but it was estimated to be approximately 45 nM by graphical interpolation. **C**. Polyadenylation assays using the wt and the mutant RNAs as substrates to test whether high-affinity RNA-binding by Nrd1p-Nab3p is required for stimulation of polyadenylation. Panels correspond to different gels.

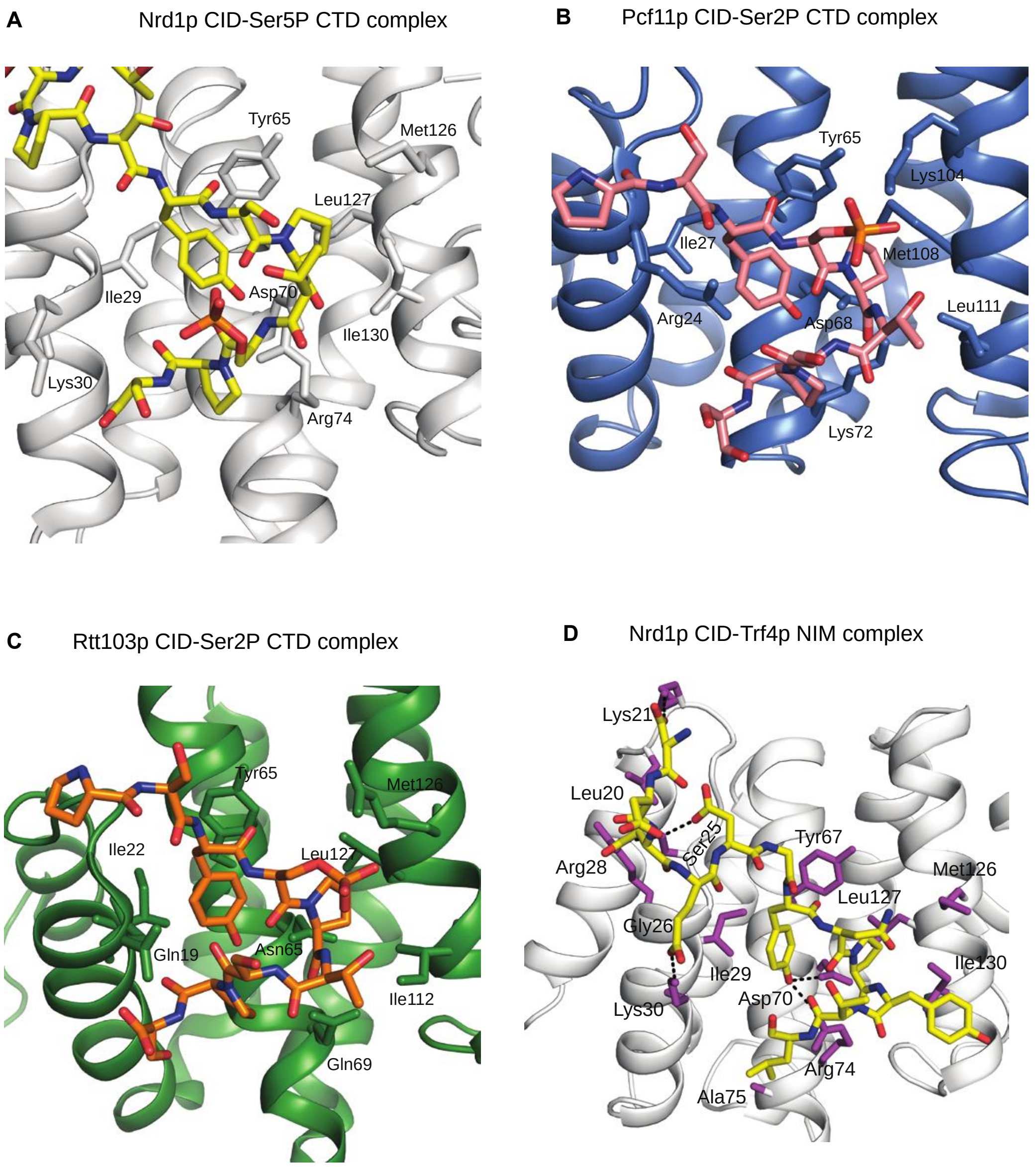

Figure S7: Related to figure 4. Comparison of different CTD-interacting domains (CIDs) bound to the phospho-CTD peptides and the NIM peptide. The comparison highlights a similar binding mode of the CTD and NIM peptides. The NIM and CTD peptides form a β-turn conformation at their (S/N)PXX motifs (regardless of the phosphorylation status) and dock into a hydrophobic pocket of CIDs. **A**. Nrd1p CID structure (white cartoon) bound to Ser5P CTD (yellow sticks; PDB ID: 2lo6). **B**. Pcf11p CID structure bound to Ser2P CTD (PDB ID: 1sza). **C**. Rtt103p CID structure bound to Ser2P CTD (PDB ID: 2l0i). **D**. Nrp1p CID structure bound to Trf4p NIM.

Supplemental experimental procedures

Construction of yeast strains

Yeast strains used in this paper are listed below. Gene deletions, tagging and insertion of the *GAL1* promoter (P_{Gal}) were performed with standard procedures (Longtine et al., 1998; Rigaut et al., 1999) using plasmids and oligonucleotides described below. Briefly, strain DLY2329 expressing untagged *nrd1ΔCID* was constructed by transforming a *Δnrd*□□strain harbouring the *URA3*-containing plasmid pRS316-Nrd1 with the product of PCR amplification using primers DL2008 and DL1993 and pRS415- *nrd1ΔCID* as the template. Cells capable of growing on 5FOA were then screened by PCR for the presence of the *nrd1ΔCID* in the chromosome and the absence of pRS414-Nrd1, and the sequence of *nrd1ΔCID* was verified by sequencing. Strain DLY2014 expressing TAP-tagged *nrd1ΔCID* used in protein interaction analyses was obtained upon isogenization of strain YSB2091 (Vasiljeva et al, 2008) by crossing it five times with BMA (YDL671).

Construction of plasmids

Plasmid pDL565 for overexpression of *TRF4-FLAG* was obtained by cleaving with *Xba*I and *Afl*III and self-ligating pETDuet-*His_6-AIR2-TRF4-FLAG* (Jia et al., 2011) to delete the fragment containing *His_6-AIR2.* Plasmid pDL567 overexpressing *His_6-AIR2* was obtained by an identical procedure but deleting the *Hin*dIII-*Xho*I fragment of pETDuet-*His_6-AIR2-TRF4-FLAG* that harbours *TRF4-FLAG.* Plasmid pDL383 was constructed by ligating the product of annealing oligonucleotides DL2043 and DL2044 into pET-Nrd1 (Vasiljeva and Buratowski, 2006) digested with *Xho*I in order to insert a TEV cleavage site between Nrd1 ORF and the His_8-tag. To obtain the variant of pDL383 expressing *nrd1Δ1-150 or nrd1ΔCID* (pDL491) the product of PCR amplification using primers DL2247 and DL2248 and *S. cerevisiae* genomic DNA as template was digested with *Pst*I and ligated into pDL383 cleaved with *Xba*I and *Pst*I. Plasmid pDL469 for overproduction of His-tagged Nab3p was constructed by amplifying *NAB3* from genomic DNA with primers DL2002 and DL2003, and ligating the corresponding *Sac*I-*Sal*I fragment of the PCR product into a pET41a-derived vector (pBS3021, kind gift of B. Seraphin) cleaved with the same enzymes. Finally, plasmid pDL613 for overexpression of *nrd1Δ332-403* (*nrd1ΔRRM*) was obtained by amplifying the corresponding *NRD1* variant from pJC606 (Conrad et al., 2000) with primers DL2000 and DL2919 and cloning the *Acc*III-*Sac*I fragment of the resulting PCR product into *Acc*III- and *Sac*I-digested pBS3021.

Plasmids for expression of mutant variants of Nrd1 CID (see the list of plasmids) were constructed using QuikChange site-directed mutagenesis kit (Stratagene, La Jolla, California).

When required for cloning purposes, the ends of DNA fragments were modified using the Klenow fragment or the T4 DNA polymerase purchased from New England Biolabs.

Analysis of RNA expression

Northern blots were performed with standard methods using 1-1.2% agarose or 5-8% polyacrylamide gels depending on the resolution range required. Hybridizations were performed using a commercial buffer (Ultrahyb, Ambion) and radiolabeled probes were prepared by random priming (Magaprime kit, GE Healthcare) or phosphorylation of oligonucleotides. Oligonucleotides used to generate probes are listed below.

DNA labeling and microarray handling

DNA samples were amplified and re-amplified with GenomePlex© Complete Whole Genome Amplification 2 (WGA2) Kit using the Farnham Lab WGA Protocol for ChIP-chip (http://www.genomecenter.ucdavis.edu/farnham/protocol.html). The re-amplification was performed in the presence of 0.4 mM dUTP (Promega U1191) to allow later enzymatic fragmentation. The enzymatic fragmentation, labeling, hybridization and array scanning were done according to the manufacturer's instructions (Affymetrix Chromatin Immunoprecipitation Assay Protocol P/N 702238). Enzymatic fragmentation and terminal labeling were performed by application of the GeneChip WT Double-Stranded DNA Terminal Labeling Kit (P/N 900812, Affymetrix). Briefly, re-amplified DNA was fragmented in the presence of 1.5 µl uracil-DNA-glycosylase (10 U/µl) and 2.25 µl APE1 (100 U/µl) at 30°C for 1 h 15 min. The fragmented DNA was then labeled at the 3′-end by adding 2 µl and 1 µl of terminal nucleotidyl transferase (TdT, 30 U/µl) and GeneChip DNA Labeling Reagent (5 mM), respectively. 5.5 µg of fragmented and labeled DNA were hybridized to a high-density custom-made Affymetrix tiling array (PN 520055) at 45°C for 16 h with constant rotational mixing at 60 rpm in a GeneChip Hybridization Oven 640 (Affymetrix, SantaClara, CA). Washing and staining of the tiling arrays were performed using the FS450_0001 script of the Affymetrix GeneChip Fluidics Station 450. The arrays were scanned using an Affymetrix GeneChip Scanner 3000 7G.

Immunoprecipitation and pull down assays

Yeast extracts were prepared by standard methods. Briefly, cell pellets were resuspended in lysis buffer (10 mM sodium phosphate pH 7, 200mM sodium acetate, 0.25% NP-40, 2mM EDTA, 1mM EGTA, 5% glycerol) containing protease inhibitors, frozen in liquid nitrogen and lysed using a Retsch MM301 Ball Mill. The protein extract was incubated with IgG Fast Flow Sepharose (GE Healthcare) and beads were washed with lysis byffer. Tagged and

associated proteins were eluted either by cleaving the protein A moiety of the tag with TEV protease in cleavage buffer (10mM Tris pH 8, 150mM NaCl, 0.1 % NP-40, 0.5mM EDTA and 1mM DTT) or by boiling the beads in 2x Laemmli buffer (100mM Tris pH 6.8, 4% SDS, 15% glycerol, 25mM EDTA, 100 mM DTT, 0.2% bromophenol blue).

For pull-down experiments each recombinant protein was overexpressed separately by growing BL21 (DE3) CodonPlus (Stratagene) cells harboring the appropriate plasmid (see the list of plasmids) on auto-inducing medium (Studier, 2005) at 20°C overnight. Protein extracts were prepared separately by sonication and subsequent centrifugation at 13000 rpm for 30 minutes at 4°C. Tagged proteins were first immobilized on Anti-FLAG affinity gel (SIGMA) or HALO link resin (Promega) and subsequently incubated with the combined bacterial extracts containing potential protein partners (typically 5-10 mg of each extract). After washing with lysis buffer the proteins were eluted by boiling the beads in 2x Laemmli buffer. When required, protein extracts were treated with 10 μg/ml of RNase A for 20 minutes at 20ºC prior to immunoprecipitation.

Chromatin immunoprecipitation (ChIP)

ChIPs were performed as previously described (Rougemaille et al., 2008). Briefly, cells grown to OD_{600} 0.8-1.0, were crosslinked with 1% formaldehyde (10 min., RT) and quenched with glycine (0.25 M final) for 5 min. Cell pellets were lyzed in FA buffer (150 mM HEPES-KOH pH 7.5, 50 mM NaCl, 1 mM EDTA, 1% Triton X-100, 1% Na deoxycholate, 0.1% SDS) containing 750 ml glass beads. Crosslinked chromatin was recovered by centrifugation and fragmented by sonication (Vibra Cell, Sonics). Immunoprecipitations were performed with either IgG Sepharose beads (GE Healthcare, for TAP-Nrd1) or the specific RNAPII CTD antibody (clone 8WG16, Merck Millipore). The bound DNA was analyzed by quantitative PCR (on 7500 Real Time PCR System (Applied Biosystems) with FastStart Universal SYBR Green master (ROX) (Roche, Basel, Switzerland). The sequences of the primers are listed below. Enrichment values were calculated relative to the DNA concentration in the extract (input). Average and standard deviation of at least three biological replicates are plotted.

Protein purification

Recombinant His-tagged Trf4-Air2 heterodimer, Nrd1p (wt and ΔCID) and Nab3p were purified from BL21 (DE3) CodonPlus harboring the appropriate pET-derived plasmid (see the list of plasmids below). Overexpression was induced by overnight growth in auto-inducing medium (Studier, 2005) at 20ºC and the protein extract was prepared by sonication of the resuspended cell pellet and subsequent clarification. Proteins were purified by Ni-affinity chromatography using a HisTrap HP 1 ml column (GE Healthcare), followed by gel filtration on a Superdex200 column pg (120 ml bed volume, GE Healthcare). The peak fractions were

dialyzed against storage buffer (20 mM sodium phosphate pH 7.5, 400 mM NaCl, 20% glycerol for Trf4-Air2 and 10 mM Tris-HCl pH 7.5, 500 mM NaCl, 50% glycerol, 1 mM DTT, for Nrd1 and Nab3) and stored at -80ºC. The Nrd1-Nab3 heterodimer was reconstituted *in vitro* by mixing the components purified separately with a 2-fold excess of Nrd1p over Nab3p and performing an additional gel filtration on a Superdex200 column to remove unbound Nrd1p.

Details on expression and purification of wt and mutant Nrd1 CID domains have been described previously (Kubicek et al., 2012).

Electromobility shift assays

RNA binding reactions were performed at room temperature in a final volume of 10 μl containing 1 nM 5'end-labeled RNA substrate and 0-100 nM recombinant Nrd1-Nab3 in polyadenylation buffer (20 mM Tris-HCl pH 7.5, 100 mM NaCl, 0.5 mM $MgCl_2$, 10% glycerol, 0.01% nonidet P-40 and 1 mM DTT). After 20-minutes incubation, 2 μl of loading buffer (0.125 % w/v bromophenol blue, 0.125% w/v xylene cyanol, 10 mM Tris HCl pH 7.5, 1 mM EDTA, 30% glycerol) were added to each reaction and the samples were subjected to electrophoresis on a 5 % polyacrylamide native gel in Tris-borate-EDTA buffer at 4ºC. Gels were processed as described for the polyadenylation assays. RNAs in the bound or the unbound fractions were quantified using the ImageQuant software (GE Healthcare) and Kd calculations were performed with Prism (GraphPad Software Inc) using a single-site binding model.

FA analyses

The equilibrium binding of Nrd1p CID to the NIM peptide and its mutants, as well as to diheptad repeat of the CTD phosphorylated at Ser5, was analyzed by fluorescence anisotropy. The CTD and Trf4-NIM peptides were N-terminally labeled with the 5,6-carboxyfluorescein (FAM; purchased from Clonestar Peptide Services, Brno, CZ). The measurements were conducted on a FluoroLog-3 spectrofluorometer (Horiba Jobin-Yvon Edison, NJ). The instrument was equipped with a thermostated cell holder with a Neslab RTE7 water bath (Thermo Scientific). The "L" format was applied using excitation and emission wavelengths of 467nm and 516nm, respectively, and each readout was averaged for 3 s. The entrance and exit slits were set to 3 nm. All measurements were performed at 10°C in 50 mM phosphate buffer (pH 8.0) containing 100 mM NaCl Each data point is an average of three measurements. The experimental binding isotherms were analyzed by non-linear least squares regression in OriginLab 7.5 software (OriginLab) using a single-site binding model to determine the binding constants (K_D).

List of yeast strains used in this work.

Number	Name	Genotype	Source
DLY17	W303	*ura3-1, ade2-1, his3-11,5, trp1-1, leu2-3,112, can1-100*	(Thomas and Rothstein, 1989)
DLY671	BMA	*as W303, ∆trp1*	F. Lacroute
DLY2348	*dis3exo-*	*dis3-D551N::ProtA::TRP1*	B. Seraphin
DLY1124	*nrd1-TAP*	*NRD1::TAP::HIS3*	T. Villa
DLY2329	*nrd1ΔCID*	*as BMA, nrd1ΔCID (Δ6-150)*	This work
DLY2014	*nrd1 ΔCID-TAP*	*as BMA, nrd1ΔCID (Δ6-150)-TAP::HIS3*	This work
DLY814	*Δrrp6*	*as BMA, rrp6::KAN*	(Porrua et al., 2012)
DLY2467	*rrp6-exoI-*	*rrp6-D238A::HIS3*	(Assenholt et al., 2008)
DLY2479	*rrp6-exoIII-*	*rrp6-Y361A::HIS3*	(Assenholt et al., 2008)
DLY2465	*rrp6-HRDC-*	*rrp6-D457A::HIS3*	(Assenholt et al., 2008)
DLY387	*Rrp41-TAP*	*as W303, RRP41::TAP::HIS3*	J. Boulay
DLY2428	*TRF4-FLAG*	*as BMA, TRF4::FLAG::TRP1(C. glabrata)*	This work
DLY2429	*trf4ΔNIM -FLAG*	*as BMA, trf4ΔNIM(Δ574-584)::FLAG::TRP1(C. glabrata)*	This work
DLY2430	*trf4ΔNIM*	*as BMA, trf4ΔNIM(Δ573-584)::TRP1(C. glabrata)*	This work
DLY2317	*P_{GAL} RRP6*	*as BMA, HIS3::P_{GAL} HA::RRP6*	This work
DLY2157	*nrd1-TAP, RRP6-myc*	*NRD1::TAP::HIS3; RRP6-myc::KAN*	This work
DLY2462	*nrd1-TAP, TRF4-FLAG*	*NRD1::TAP::HIS3; TRF4::FLAG::TRP1(C. glabrata)*	This work
DLY2463	*nrd1-TAP, trf4ΔNIM -FLAG*	*as BMA, NRD1::TAP::HIS3; trf4ΔNIM::FLAG::TRP1(C. glabrata)*	This work
DLY2041	*nrd1 ΔCID-TAP, rrp6-myc*	*as BMA ,nrd1ΔCID::TAP::HIS3; RRP6-myc::KAN*	This work
DLY2336	*nrd1ΔCID, Δrrp6*	*as BMA, nrd1ΔCID; rrp6::KAN*	This work
DLY2467	*nrd1ΔCID, rrp6-exoI-*	*nrd1ΔCID; rrp6-D238A*	This work
DLY2479	*nrd1ΔCID, rrp6-exoIII-*	*nrd1ΔCID; rrp6-Y361A*	This work
DLY2465	*nrd1ΔCID, rrp6-HRDC-*	*nrd1ΔCID; rrp6-D457A*	This work
DLY2480	*trf4ΔNIM, dis3exo-*	*as BMA ,trf4ΔNIM ::TRP1(C. glabrata); dis3-D551N::ProtA::TRP1*	This work
DLY2360	*P_{GAL} RRP6, Δupf1*	*as BMA, HIS3::P_{GAL} HA::RRP6; upf1::TRP1*	This work
DLY2337	*P_{GAL} RRP6, nrd1 ΔCID*	*as BMA, HIS3::P_{GAL} HA::RRP6; nrd1ΔCID*	This work
DLY2361	*P_{GAL} RRP6, Δupf1, nrd1 ΔCID*	*as BMA, HIS3::P_{GAL} HA::RRP6; upf1::TRP1; nrd1 ΔCID*	This work
DLY1680	*NRD1-VSV*	*as BMA, NRD1::VSV::KAN*	This work
DLY2403	*Rrp41-TAP, Δrrp6, NRD1-VSV*	*as W303, RRP41::TAP::HIS3; rrp6::KAN, NRD1::VSV::KAN*	This work
DLY2404	*Rrp41-TAP, Δtrf4, NRD1-VSV*	*RRP41::TAP::HIS3; trf4::KAN MX, NRD1::VSV::KAN*	This work
DLY1636	*sen1-2*	*ade2-101, his3-200, lys2-801, trp1∆1, ura3-52, leu2∆1::sen1-2::LEU2, sen1::TRP1*	(Finkel et al., 2010)

List of plasmids used in this work.

Name	Description	Source
pFA6a-His3MX6-PGAL1-3HA	Ap[r], *ori*ColE1; plasmid bearing the *HIS3* P_{GAL}*::HA* tagging cassette	(Longtine et al., 1998)
pBS2215	Ap[r], *ori*ColE1; plasmid bearing cassette for C-terminal tagging with *CBP::U1A::TRP1(C. glabrata)*	(Finoux and Séraphin, 2006)
pDL383	Km[r]; *ori*ColE1; derivative of pET41a bearing yeast *NRD1-TEVcl*-His*$_8$ under the control of the T7 promoter.	This work
pDL469	Km[r]; *ori*ColE1; derivative of pET41a bearing yeast *NAB3-TEVcl-His*$_6$ under the control of the T7 promoter	This work
pDL491	Km[r]; *ori*ColE1; derivative of pET41a bearing yeast *nrd1ΔCID- TEVcl-His*$_8$ under the control of the T7 promoter.	This work
pET15b-His$_6$-MTR4	Ap[r], *ori*ColE1; plasmid expressing *His*$_6$*-MTR4* from the T7 promoter	(Wang et al., 2008)
pETDuet-His$_6$-AIR2- TRF4-FLAG	Ap[r], *ori*ColE1; derivative of pETDuet bearing *His*$_6$*-AIR2* and *TRF4-FLAG* under the control of the T7 promoter.	(Jia et al., 2011)
pDL565	Ap[r], *ori*ColE1; derivative of pETDuet bearing *TRF4-FLAG* under the control of the T7 promoter.	This work
pDL567	Ap[r], *ori*ColE1; derivative of pETDuet bearing *His*$_6$*-AIR2* under the control of the T7 promoter.	This work
pBS3936	Ap[r]; *ori*ColE1; plasmid bearing *Rrp6-HALO* under the control of the T7 promoter	B. Seraphin
pDL613	Km[r]; *ori*ColE1; derivative of pET41a bearing yeast *nrd1ΔRRM-TEVcl-His*$_6$ under the control of the T7 promoter.	This work
pU6H3VSV	Ap[r]; Km[r]; *ori*ColE1; plasmid containing *6His::3VSV::loxP::kanMX::loxP* cassette for C-terminus epitope tagging.	(De Antoni and Gallwitz, 2000)
pRS_NC	Ap[r]; *ori*ColE1; plasmid bearing CID-His$_6$ under the control of the T7 promoter	(Kubicek et al., 2012)
pRS_NC_L20D_	Kan[r]; *ori*ColE1; plasmid bearing CID(L20D)-His$_6$ under the control of the T7 promoter	(Vasiljeva et al., 2008)
pRS_NC_K21D_	Kan[r]; *ori*ColE1; plasmid bearing CID(K21D)-His$_6$ under the control of the T7 promoter	(Vasiljeva et al., 2008)
pRS_NC_S25R_	Ap[r]; *ori*ColE1; plasmid bearing CID(S25R)-His$_6$ under the control of the T7 promoter	(Kubicek et al., 2012)
pRS_NC_R28D_	Ap[r]; *ori*ColE1; plasmid bearing CID(R28D)-His$_6$ under the control of the T7 promoter	(Kubicek et al., 2012)
pRS_NC_D70A_	Ap[r]; *ori*ColE1; plasmid bearing CID(D70A)-His$_6$ under the control of the T7 promoter	This study
pRS_NC_I130K_	Ap[r]; *ori*ColE1; plasmid bearing CID(I130K)-His$_6$ under the control of the T7 promoter	This study

*TEV cleavage site

List of oligonucleotides used in this work.

Name	Sequence (5' to 3')	Description/use
DL377	ATGTTCCCAGGTATTGCCGA	Fwd primer to generate an *ACT1* probe
DL378	ACACTTGTGGTGAACGATAG	Rev primer to generate an *ACT1* probe
DL474	GCAAAGATCTGTATGAAAGG	Rev primer to generate a NEL025c probe
DL478	CCTGTTGACATTGCAGACAA	Fwd primer to generate a NEL025c probe
DL1566	AGTTGATCGGACGGGAAAC	Rev to 5S rRNA to generate an oligonucleotide probe
DL1120	TCCGTGTCTCTTGTCCTGCA	Rev primer to generate a snR13 probe
DL1452	CTTCCCCGTAGAAAATCTTA	Fwd primer to generate a snR13 probe
DL1154	CCTATAACAACAACAACATG	Fwd primer to generate a snR47 probe
DL1157	ATAGCCATTAGTAAGTACGC	Rev primer to generate a snR47 probe
DL1687	GGTTGTTTGGCCGAGCGGTC	Fwd primer to generate a probe to detect tRNA Leu (SUP53)
DL417	TGGTTGCTAAGAGATTCGAAC	Rev primer to generate a probe to detect tRNA Leu (SUP53)
DL1694	ATCCTTGCTTAAGCAAATGCGCTTAAAAGCCGAACGCT<br>CTACCAACTGAGCTAA	Rev. to tRNA Lys (tK(UUU)D)) – oligo Northern probe
DL262	ATCCTTATGCACGGGAAA	Fwd primer to generate an U4 probe
DL263	CACCGAATTGACCATGAG	Rev primer to generate an U4 probe
DL2963	GGCCCACAGTTCTTGATGTTGACCTCCCTCCGCCATT<br>GATC	Rev to U5 (snR14) to generate an oligonucleotide probe
DL2637	TAGACGAAATAGGAACAACAAACAGCTTATAAGCACCC<br>AATAAGTGCGTTGAATTCGAGCTCGTTTAAAC	Fwd primer to construct a *PGAL-RRP6* strain
DL2638	ACCACATTTATCACCCTAGATAAAAGTACATCCGGATT<br>TTCAGAAGTCATGCACTGAGCAGCGTAATCTG	Rev primer to construct a *PGAL-RRP6* strain
DL2892	GTACACAGTGATGTACAGTTCAGTGCATCATTTAAACA<br>AAAAGGCACATATACGACTCACTATAGGGCGA	Rev primer to construct: *TRF4 ΔNIM*; *TRF4-FLAG* and *TRF4 ΔNIM -FLAG* strains
DL2893	AAGATGATGATGAAGATGGATATAATCCTTATACCCTT<br>GACTACAAGGACGACGATGACAAATAAGGATCCGTCG<br>ACCAAGC	Fwd primer to construct a*TRF4-FLAG* strain
DL2894	GAAATAGTGGAGAGACATATATCACTGTCTCTAGCGAA<br>GACTACAAGGACGACGATGACAAATAAGGATCCGTCG<br>ACCAAGC	Fwd primer to construct a *TRF4ΔNIM -FLAG* strain
DL2897	CGGCCAAGAGAAATAGTGGAGAGACATATATCACTGT<br>CTCTAGCGAATAAGGATCCGTCGACCAAGCTTG	Fwd primer to construct a *TRF4ΔNIM* strain
DL2043	TCGACGAAAACCTGTATTTTCAGGGAG	Fwd oligonucleotide encoding a TEV cleavage site
DL2044	TCGACTCCCTGAAAATACAGGTTTTCG	Rev oligonucleotide encoding a TEV cleavage site
DL2002	ACGTTTGAGCTCAATAATTTTGTTTAACTTTAAGAAGGA<br>GTGCAAGCCATGTCAGATGAAA	Fwd primer to construct pDL469 (pET41a- *NAB3-TEVcl-*His_6)
DL2003	TACAAGGTCGACTCAGTGGTGGTGGTGGTGGTGTCCC<br>TGAAAATACAGGTTTTCTTTTTGTAGTTTTGCTAAACTA	Rev primer to construct pDL469 (pET41a- *NAB3-TEVcl-*His_6)
DL2008	TAGTACTTTTCTCCAAGCAC	Fwd primer at position -70 relative to NRD1 start codon
DL1993	ACCAGGAATACGGTAA	Rev primer at position +189 relative to NRD1 stop codon
DL2247	AGAGCTTCCGGAAATAATTTTGTTTAACTTTAAGAAGG<br>AGATCCCATAATGGCTATGGACATATCGAATAA	Fwd primer to construct pDL491 (pET41a- *nrd1ΔCID-TEVcl-*His_8)
DL2248	AGGTGCGACTGTACCCACGG	Rev primer to construct pDL491 (pET41a- *nrd1ΔCID-TEVcl-*His_8)
DL2000	AGAGCTTCCGGAAATAATTTTGTTTAACTTTAAGAAGG<br>AGATCCCATAATGCAGCAGGAC	Fwd primer to construct pDL613 (pET41a- *nrd1ΔRRM-TEVcl-*His_6)
DL2919	TACAAGGAGCTCTCAGTGGTGGTGGTGGTGGTGTCCC<br>TGAAAATACAGGTTTTCGCTTTGTTGTTGTTGCTGCT	Rev primer to construct pDL613

DL2974	GAAACGUAAUGAAUUAAGUCUUGAUAUAUAACAAUUAGCU	RNA substrate for polyadenylation assays (Wt RNA)
DL2975	GAAACGCAAUGAAUUAAGACUCGAUACAUUGCAAUUAGCU	RNA substrate for polyadenylation assays (Mutant RNA)
DL1117	GAGTGCATTTGGCTCGAGTTGC	Fwd primer for ChIP-qPCR to amplify *snR13* body
DL1118	TTCCACACCGTTACTGATTT	Rev primer for ChIP-qPCR to amplify *SNR13* body
DL2959	CTCCTAGTTCTCTTCCCAGAGCAT	Fwd primer for ChIP-qPCR to amplify *SNR13* read-through region
DL2960	AAGTCGCTGTGCTGGAGTTAG	Rev primer for ChIP-qPCR to amplify *SNR13* read-through region
DL474	GCAAAGATCTGTATGAAAGG	Fwd primer for ChIP-qPCR to amplify *NEL025c* body
DL475	CGCAGAGTTCTTACCAAACG	Rev primer for ChIP-qPCR to amplify *NEL025c* body
DL483	TCAGCACAGAACGTAACGAC	Fwd primer for ChIP-qPCR to amplify *NEL025c* read-through region
DL484	CAATTTTTGAGCCCACATGC	Rev primer for ChIP-qPCR to amplify *NEL025c* read-through region
RS_NC_D70A_f	CATCGCCTCAATAGGTAGAGCTTACTTGGA TGAAAC	Fwd primer for PIPE mutagenesis of D70A
RS_NC_D70A_r	GTTTCATCCAAGTAAGCTCTACCTATTGAGGCGATGATATATAAAG	Rev primer for PIPE mutagenesis of D70A
RS_NC_I130K_f	GCTTTTAGACAAGTGGGACAGGTCCGGCTTGTTTCAAAAGAGTTAC	Fwd primer for PIPE mutagenesis of I130K
RS_NC_I130K_r	CCTGTCCCACTTGTCTAAAAGCATACGAATTTTTTCCTTGTGGTCTTG	Rev primer for PIPE mutagenesis of I130K

Supplemental References

De Antoni, A., and Gallwitz, D. (2000). A novel multi-purpose cassette for repeated integrative epitope tagging of genes in Saccharomyces cerevisiae. Gene *246*, 179–185.

Assenholt, J., Mouaikel, J., Andersen, K.R., Brodersen, D.E., Libri, D., and Jensen, T.H. (2008). Exonucleolysis is required for nuclear mRNA quality control in yeast THO mutants. RNA N. Y. N *14*, 2305–2313.

Conrad, N.K., Wilson, S.M., Steinmetz, E.J., Patturajan, M., Brow, D.A., Swanson, M.S., and Corden, J.L. (2000). A yeast heterogeneous nuclear ribonucleoprotein complex associated with RNA polymerase II. Genetics *154*, 557–571.

Finkel, J.S., Chinchilla, K., Ursic, D., and Culbertson, M.R. (2010). Sen1p performs two genetically separable functions in transcription and processing of U5 small nuclear RNA in Saccharomyces cerevisiae. Genetics *184*, 107–118.

Finoux, A.-L., and Séraphin, B. (2006). In vivo targeting of the yeast Pop2 deadenylase subunit to reporter transcripts induces their rapid degradation and generates new decay intermediates. J. Biol. Chem. *281*, 25940–25947.

Longtine, M.S., McKenzie, A., 3rd, Demarini, D.J., Shah, N.G., Wach, A., Brachat, A., Philippsen, P., and Pringle, J.R. (1998). Additional modules for versatile and economical PCR-based gene deletion and modification in Saccharomyces cerevisiae. Yeast Chichester Engl. *14*, 953–961.

Phillips, S., and Butler, J.S. (2003). Contribution of domain structure to the RNA 3’ end processing and degradation functions of the nuclear exosome subunit Rrp6p. RNA N. Y. N *9*, 1098–1107.

Rigaut, G., Shevchenko, A., Rutz, B., Wilm, M., Mann, M., and Séraphin, B. (1999). A generic protein purification method for protein complex characterization and proteome exploration. Nat. Biotechnol. *17*, 1030–1032.

Rougemaille, M., Dieppois, G., Kisseleva-Romanova, E., Gudipati, R.K., Lemoine, S., Blugeon, C., Boulay, J., Jensen, T.H., Stutz, F., Devaux, F., et al. (2008). THO/Sub2p functions to coordinate 3’-end processing with gene-nuclear pore association. Cell *135*, 308–321.

Studier, F.W. (2005). Protein production by auto-induction in high density shaking cultures. Protein Expr. Purif. *41*, 207–234.

Thomas, B.J., and Rothstein, R. (1989). The genetic control of direct-repeat recombination in Saccharomyces: the effect of rad52 and rad1 on mitotic recombination at GAL10, a transcriptionally regulated gene. Genetics *123*, 725–738.

Wang, X., Jia, H., Jankowsky, E., and Anderson, J.T. (2008). Degradation of hypomodified tRNA(iMet) in vivo involves RNA-dependent ATPase activity of the DExH helicase Mtr4p. RNA N. Y. N *14*, 107–116.
